# Association of Social Needs and Housing Status Among Urban Emergency Department Patients

**DOI:** 10.5811/westjem.2022.8.55705

**Published:** 2022-10-28

**Authors:** Kadia Wormley, Drusia Dickson, Harrison Alter, Ndidi Njoku, Partow Imani, Erik S. Anderson

**Affiliations:** * Department of Emergency Medicine, Alameda Health System, Oakland, California; † Andrew Levitt Center for Social Emergency Medicine, Berkeley, California; ‡ Howard University College of Medicine, Washington, DC; § University of California Berkeley, School of Public Health, Berkeley, California; ¶ Substance Use Disorder Treatment Program, Alameda Health System, Oakland, California

## Abstract

**Introduction:**

People experiencing homelessness have high rates of social needs when presenting for emergency department (ED) services, but less is known about patients with housing instability who do not meet the established definitions of homelessness.

**Methods:**

We surveyed patients in an urban, safety-net ED from June–August 2018. Patients completed two social needs screening tools and responded to additional questions on housing. Housing status was determined using validated questions about housing stability.

**Results:**

Of the 1,263 eligible patients, 758 (60.0%) completed the survey. Among respondents, 40% identified as Latinx, 39% Black, 15% White, 5% Asian, and 8% other race/ethnicities. The median age was 42 years (interquartile range [IQR]: 29–57). and 54% were male. Of the 758 patients who completed the survey, 281 (37.1%) were housed, 213 (28.1%) were unstably housed, and 264 (34.8%) were homeless. A disproportionate number of patients experiencing homelessness were male (63.3%) and Black (54.2%), P <0.001, and a disproportionate number of unstably housed patients were Latinx (56.8%) or were primarily Spanish speaking (49.3%), P <0.001. Social needs increased across the spectrum of housing from housed to unstably housed and homeless, even when controlling for demographic characteristics.

**Conclusion:**

Over one in three ED patients experience homelessness, and nearly one in three are unstably housed. Notable disparities exist by housing status, and there is a clear increase of social needs across the housing spectrum. Emergency departments should consider integrating social screening tools for patients with unstable housing.

## INTRODUCTION

Homelessness is a well-established factor associated with poor health outcomes. People experiencing homelessness (PEH) have higher mortality and morbidity than the general population,^1^–^8^ as well as higher incidences of substance use disorders and mental illness.^9^–^15^ The majority of adults experiencing homelessness lack a regular source of healthcare.^1^,^6^ They face numerous barriers to accessing care including lack of insurance, financial limitations, lack of transportation, difficulty making appointments, stigma, and competing immediate needs such as food and shelter.^16^ Additionally, there are significant racial and ethnic disparities, with communities of color disproportionately impacted by homelessness.^17^

For all these reasons, the emergency department (ED) is a major purveyor of healthcare for PEH.^18^ This touch point within the healthcare system is recognized as an important opportunity to address housing instability and social needs, as evidenced by the passage of California State Senate bill 112, which requires hospitals to identify PEH and offer specific resources prior to discharge including food, shelter, and transportation.^19^ As there is no funding attached to the bill, California EDs have attempted to address the requirements of SB 1152 variably and have largely modified documentation of existing resources for PEH. There is, however, a large body of literature that documents the complex social needs of PEH and ED-based interventions developed to improve outcomes in this population.^20^

The spectrum of housing also includes housing instability, which does not have a standard definition in the healthcare literature.^21^ It is variably referred to as housing instability, housing insecurity, unstable housing, marginal housing, housing vulnerability and is sometimes grouped together with homelessness as the umbrella term “homeless and unstably housed.” These terms refer to a range of experiences contributing to a precarious living situation, including difficulty paying rent or mortgage; spending the majority of monthly income on rent; living in crowded spaces; living with others for free; being evicted; or moving frequently.^22^

Perhaps because of its lack of clear definition, housing instability and its effect on health has been less well studied than homelessness. Both populations have increased rates of unmet basic healthcare needs,^3^ violence,^23^ human immunodeficiency virus and hepatitis C virus,^24^ and overall mortality.^25^,^26^ Prior studies have also shown associations between housing instability and anxiety and depression,^27^ increased substance abuse and psychiatric symptoms,^28^ poorer access to healthcare,^29^ and high rates of acute care use.^30^ Unstably housed persons have increased social needs compared to stably housed persons of similar income, suggesting that housing insecurity is a graded risk factor, with patients experiencing worse health outcomes as housing instability increases.^29^

It is likely that unstable housing and homelessness are underrecognized, despite their high prevalence among ED patients.^18^ People experiencing housing instability are at high risk of becoming homeless,^31^ yet little is known about this population in the ED.

### Study Aim

Our goal in this study was to compare the demographics and social needs of patients presenting to an urban ED stratified by housing status.

## METHODS

### Study Design

We conducted a cross-sectional study of patients from an urban, safety-net ED and Level I trauma center in Oakland, California, with 68,000 annual visits. All patients ≥18 years who spoke English or Spanish and presented to the ED during study hours were considered eligible. We excluded minors because our ED sees only a small number of pediatric patients. Patients were also excluded if they were medically unstable, unresponsive, had altered mental status precluding participation, or had already participated in the study. The study was approved by the institutional review board at Alameda Health System.

**Population Health Research Capsule**

What do we already know about this issue?

*Despite the detrimental effect of housing insecurity on health outcomes, the prevalence of homelessness and housing insecurity is likely underrecognized in EDs.*

What was the research question?

*What are the demographics and social needs of patients presenting to an urban ED stratified by housing status?*

What was the major finding of the study?

*Over 1/3 of patients experience homelessness, nearly 1/3 are unstably housed, and social needs rose across this housing spectrum.*

How does this improve population health?

*We highlight the burden of housing insecurity and associated social needs among urban ED patients. Our findings suggest opportunities for ED-based interventions.*

### Survey Development

Survey administration, development, and validation is described in a prior manuscript.^32^ The survey instrument used questions from two social needs screening tools: the Protocol for Responding to and Assessing Patient Assets, Risks, and Experiences (PRAPARE), developed by the National Association of Community Health Centers,^33^ and the Accountable Health Communities (AHC) Health-Related Social Needs Screening Tool, developed by the Centers for Medicare and Medicaid Services.^34^ The full survey instrument is available in Appendix A.

### Housing Categories

We divided respondents into three housing categories: homeless, unstably housed, and stably housed. The questions defining each category were selected from the two surveys mentioned above with additional questions developed by an expert committee to better understand our population’s housing status. In accordance with standard definitions of homelessness, patients were considered to be experiencing homelessness if they responded “Yes” to any of the following statements: “I do not have housing;” “I do not have a steady place to live;” “I am currently homeless;” or “Last night I stayed at a shelter, housing for homeless persons, a location not meant for human habitation, or a friend/family member’s room/apartment.”

Patients were considered unstably housed if they answered “Yes” to any of the following statements: “I am worried about my housing”; “I have a place to stay, but I am worried about losing it”; “I have moved three or more times in the last 12 months”; “I had to move in with other people in the last 12 months because of housing problems”; or “I am unable to stay in current place for more than 90 days.” If patients answered “No” to all statements, they were considered to be stably housed.

### Survey Administration and Data Abstraction

Patients were recruited in four-hour blocks of time covering all times of day, for a total of two full weeks (14 days, 24 hours/day) between June–August 2018. Trained research assistants (RA) approached patients during their ED visit and obtained verbal consent using a standardized script. The RAs systematically approached patients in order of arrival time and, when possible, returned to patients who were unavailable at the time of the initial approach. During study blocks, RAs were not able to approach every eligible patient who was registered due to time constraints. Eligible patients who were not approached were included in an analysis of non-respondents.

Using a password-protected tablet, survey responses from participants were input directly into REDCap, a secure electronic data capture system^35^,^36^ hosted at Alameda Health System. The RAs read the questions aloud or participants completed the survey directly on the tablet; RAs were bilingual Spanish and English speakers. We excluded non-English or Spanish speakers as the hospital interpreters were not available for research purposes. Trained abstractors documented arrival and discharge times, disposition, medical history, prior ED utilization, and past admissions from the electronic health record (EHR) (Wellsoft Corporation, Somerset, NJ) during a standardized chart review.

### Outcomes

The primary outcomes were the proportion of homeless, unstably housed, and stably housed patients in our cohort. Secondary outcomes included demographics and social needs among patients in each housing category. We also used regression analysis to control for demographic characteristics to explore the graded risk of social needs along the housing spectrum.

### Data Analysis

For each housing category, we calculated standard descriptive statistics. We reported continuous variables as medians and means and reported categorical variables as proportions or percentages. We made comparisons by using chi-square, ANOVA, and Mann-Whitney tests between outcome variables. We considered *P* <.05 to be significant for comparisons between data points.

For all individuals without any missing values (n = 714), we used a separate logistic regression for each social factor, where the social factor was regressed on housing status as well as adjusting for the following covariates: age; gender; race/ethnicity; education; primary language; English proficiency; veteran status; insurance; disability; and past medical history. The outcomes were assumed to be conditionally linear in their relationship to housing status with the link function. The estimated coefficient was associated with housing status for all 17 regressions. In addition, a permutation test was performed where over 500 iterations, the housing status variable was randomly shuffled, thereby breaking any association between housing status and the various outcomes of interest. The regressions were again used in each of the 500 iterations, and we compared the observed statistics from the un-permuted data to the null distribution created by the random permutations.

We performed a propensity score analysis using the EHR to determine whether the survey respondents were substantively different from patients who were potentially eligible but did participate in the survey. We included patients who were approached but declined to participate, as well as potentially eligible patients who were not approached. If patients were ineligible once approached (did not speak English or Spanish, had altered mental status, or were critically ill), they were not included in the analysis of non-respondents. Respondents were randomly selected and paired 1:1 with non-respondents matched by hour of arrival. The propensity score analysis included the following covariates: age; gender; acuity; language; race; insurance type; disposition; past medical history; whether the patient was on a psychiatric hold or in legal custody; homelessness documented in the chart; and ED and hospital admissions in the 12 months prior to study visit. We performed analyses using R Core Team (2017) (R Foundation for Statistical Computing, Vienna, Austria) and Stata version 15.1 (StataCorp LLC, College Station, TX). Incomplete surveys were not included in the analyses.

## RESULTS

During the study period, there were 2,573 ED visits from 2,357 unique patients. Of these, 1,522 patients were approached and screened for survey administration, and 1,263 were deemed eligible. Of the 1,263 eligible patients, 758 (60.0%) completed the survey, 478 declined, and 27 started but did not complete the survey. Among respondents, 40% identified as Latinx, 39% Black, 15% White, 5% Asian, and 8% other race/ethnicities. The median age was 42 years (interquartile range [IQR]: 29–57) and 54% were male.

Of the 758 patients who completed the survey, 281 (37.1%) were housed, 213 (28.1%) were unstably housed, and 264 (34.8%) were homeless. There were significant differences across all demographic variables analyzed by housing status (Table 1) other than veteran status. Notable disparities in demographic characteristics by housing category compared to the study population as a whole included the following: a higher proportion of patients aged 25–54 years who were unstably housed (68.1% vs 57.0%); male patients experiencing homelessness (63.3% vs 54.1%); Black patients experiencing homelessness (54.2% vs 38.8%), Latinx patients who were unstably housed (56.8% vs 40.2%), and Spanish-speaking patients who were unstably housed (49.3% vs 28.5%). Thirty-five (13.3%) of the 264 PEH in our study had homeless or housing instability noted in the chart, and only one (0.4%) of the unstably housed patients had any housing instability documented in their EHR.

The healthcare utilization of patients by housing status was notable for a higher median number of ED visits in the 12 months preceding the study among PEH (median 2, IQR: 2–5), compared to unstably housed (median 2, IQR: 1–3) and housed patients (median 2, IQR: 1–3), *P* = 0.02. There were no differences in hospitalization rates by housing category in the year prior to survey administration (Table 2). We found significant differences in disposition from the study ED visit by housing category at the index visit, however with higher rates of admission among housed patients (14.2%) compared to unstably housed (7.0%) and PEH (7.6%), and higher rates of disposition to psychiatric facilities among patients experiencing homelessness (3.4%) compared to unstably housed (0.0%) and housed patients (0.1%), *P* <0.001. More homeless patients (4.5%) were in custody at the time of their ED visit compared to unstably housed (1.4%) and housed patients (1.1%), *P* < 0.02.

Table 3 shows the social, emotional, and substance use needs of patients by housing category. Across each category of social needs, emotional stress and trauma, and substance use history, the prevalence increased across the housing spectrum, with housed being the lowest, followed by unstably housed, followed by homeless with the highest prevalence.

**Figure 1:**
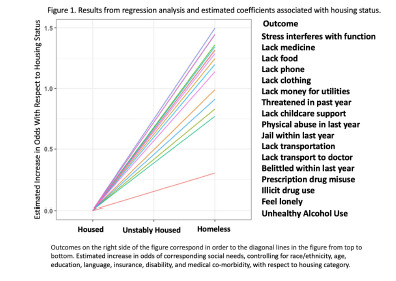
Results from regression analysis and estimated coefficients associated with housing status.

We reported the estimated coefficient associated with housing status for all 17 regressions, and the resulting lines are visualized in Figure 1. Each social factor was associated with increased risk as patients progressed from housed to unstably housed, with the highest risk for PEH. The regressions were again used in each of the 500 iterations, and the observed coefficient statistics compared to the null distribution created by the random permutations, which can be seen in Appendix B. When randomly inserting housing status, the distribution of coefficients for all of the social needs variables were significantly different than the observed coefficient, indicating a significant association with housing status for all of the analyzed social needs.

The full results of the propensity score analysis were published in a prior manuscript; the distribution of scores grouped toward the middle suggested that the respondents and non-respondents were similar with regard to baseline characteristics.^32^

## DISCUSSION

We found that the majority of patients in our study faced homelessness acutely or imminently, with 37% of ED patients experiencing homelessness and 28% who were unstably housed. This is a much higher prevalence than in previous ED studies.^13^,^37^,^38^ This higher prevalence is likely explained by several factors, some of which are unique to our ED and part of the country. Our study takes place in an urban safety-net ED in a geographic region that has high rates of homelessness and housing instability. It is important to note that while this may be a finding that may not be applicable to all EDs, the high rates of housing instability and social needs among patients in our ED highlights the important role of safety-net EDs for vulnerable communities. Given the stark disparities in the US healthcare system, our work is likely generalizable to many EDs serving similar populations, but the findings may be less informative for EDs serving more privately insured patients or in parts of the country with lower rates of homelessness. Moreover, the observation of a graded risk of housing associated with increasingly prevalent social needs suggests that developing ED-based interventions for patients who are unstably housed may be particularly important areas for future work.

To intervene on behalf of these particularly vulnerable patients, we must first recognize and identify them. There was a large discrepancy between the housing category identified in the study and what was documented in the study participants’ corresponding medical charts: <1% in the unstably housed group and 13% in the homeless group had documentation in the EHR correctly reflecting their housing status. Screening for housing instability is lacking in most EDs, and screening tools to ask about housing instability, perhaps by including the questions used in this study, could be integrated into ED-based screening programs.^32^,^33^ Additional questions could prove somewhat burdensome for many EDs without proper support, and further investigation is needed to confirm the optimal number and combination of questions to screen for housing insecurity.

We found notable demographic disparities in patients with unstable housing compared with PEH in our population. Housing insecurity and homelessness have been shown to affect people of color at vastly disproportionate rates, with Black populations estimated to be four times as likely to experience homelessness during their lifetime than their White counterparts and Latinx twice as likely.^17^ In our cohort, Latinx patients were disproportionately overrepresented in the unstably housed group. Additionally, patients who were unstably housed were more likely to report a significant disability (22%) compared to PEH (4.5%) and stably housed individuals (12.1%). This is consistent with other data showing that US poverty rates among those with disabilities is more than twice as high as those without.^39^ Unstably housed patients also reported lower levels of English proficiency or speaking a primary language other than English, suggesting a higher immigrant population in this group. Research strongly suggests that language barriers adversely affect patients’ health status and ability to access healthcare, although less is known about the impact of language on housing stability.^40^,^41^

Given that housing instability is a graded risk factor, and that there are known poor outcomes for PEH,^6^ unstably housed populations are a prime target for harm-reduction interventions. Interventions in the ED could target a specific social need, like food insecurity (present in 27% of unstably housed individuals in our study), or specific social needs most prevalent in a particular community. Case management or other approaches to ensure that patients who are unstably housed do not “fall through the cracks” regarding their social needs could help lessen stressors and possibly prevent progression to homelessness. By identifying and targeting this vulnerable group, ED-based interventions could be targeted to have significant impact on patient outcomes and address needs of patients who are unstably housed before progression to homelessness.

In our ED we have attempted to address social needs holistically, rather than attempting to take on the entirety of a patient’s housing needs from a brief ED visit. Realistically, finding permanent supportive housing is extremely complicated, and is an unreasonable expectation to place on emergency clinicians. Rather, we have modified our approach to target specific needs of our population who are experiencing homelessness or are unstably housed. We do have a general approach to PEH that includes a partnership with social work and local housing organizations, but it is often more practicable to address individual needs. While this approach may only be related to some of the underlying social issues, EDs should consider addressing some of the specific needs of patients given the complexities of the housing crisis — especially in urban areas with large homeless and unstably housed populations. For example, our social work and substance use disorder treatment teams routinely work to provide PEH and unstably housed patients with food and clothing, thereby integrating individual needs while seeking temporary emergency shelter placement if patients are agreeable. Additionally, our approach to these interventions is specifically trauma informed; support staff all receive training in trauma-informed care, helping us to also consider the past trauma, psychosocial, and emotional needs of our patients when addressing social determinants of health.

The consistent increase in social needs as patients progressed from housed, to unstably housed, to homeless is in line with studies showing that housing stability is a graded risk factor for poorer outcomes among populations outside the ED.^29^,^42^ More research is needed regarding the benefits of ED screening for housing instability, but neglecting to screen for and target the unstably housed, and focusing solely on homelessness, is similar to ignoring angina and only treating the acute heart attack: a missed opportunity for intervention and risk reduction.

## LIMITATIONS

Our study has several important limitations. This data represents a single-center, convenience sample in an urban setting and may not be generalizable to EDs in other settings. There are seasonal variations to homelessness and because our study was conducted in summer months, data may not be representative of housing statistics at other times of the year. Further, only 65% of all patients eligible during study periods were approached. This was mostly due to limited time capacity of RAs, which may have biased who was approached.^32^ This data notably includes patients in custody at time of the survey, who are excluded from federal definitions of homelessness. It does not include data from patients who presented medically unstable or unresponsive, or who were unable to complete the survey due to initiation of medical care. It’s possible that the sicker patients who were excluded by this study design had even higher levels of homeless and housing instability, given what we know about PEH having a higher burden of illness and mortality.

Another limitation was that surveys were only conducted in English and Spanish, with 17% of screened patients ineligible due to a language barrier. Finally, there is no standard definition of housing instability. As discussed, we made our own screening tool and used a more comprehensive definition than prior studies. The question of how to define and identify housing instability remains central to further work in this area.

## CONCLUSION

In our study sample we found nearly one third of our patient population was unstably housed, and another third was experiencing homelessness. We note important disparities, including higher rates of homelessness among Black patients, and higher rates of unstable housing among Latinx and Spanish-speaking patients. We also found that social, emotional, and substance abuse-related needs increased significantly as housing became more unstable, even when controlling for baseline demographic characteristics.

## Supplementary Information





## Figures and Tables

**Table 1:** Baseline characteristics of all respondents by housing status.

Sociodemographic characteristics	Overall N = 758		Housed N = 281 (37.1%)		Unstably housed N = 213 (28.1%)		Homeless N = 264 (34.8%)		*P* value
Age group									***P*** ** <0.001**
18 – 24 years	100	13.2%	44	15.7%	20	9.4%	36	13.6%	
25 – 54 years	439	57.9%	139	49.5%	145	68.1%	155	58.7%	
55 – 64 years	138	18.2%	55	19.6%	32	15.0%	51	19.3%	
> 64 years	81	10.7%	43	15.3%	16	7.5%	22	8.3%	
Male	410	54.1%	130	46.3%	113	53.1%	167	63.3%	***P*** ** < 0.001**
Race/Ethnicity									***P*** ** < 0.001**
Black/African American	294	38.8%	97	34.5%	54	25.4%	143	54.2%	
Latinx	305	40.2%	119	42.3%	121	56.8%	65	24.6%	
White	112	14.8%	44	15.7%	29	13.6%	39	14.8%	
Asian	39	5.1%	18	6.4%	7	3.3%	14	5.3%	
Other	59	7.8%	23	8.2%	10	4.7%	26	9.8%	
Education									***P*** ** < 0.001**
Less than a high school degree	210	27.7%	61	21.7%	83	39.0%	66	25.0%	
High school diploma or GED	260	34.3%	97	34.5%	55	25.8%	108	40.9%	
More than high school	281	37.1%	122	43.4%	73	34.3%	86	32.6%	
Median Income (IQR)			20,000	11,000–45,000	18,000	10,000–28,500	11,000	1,000–21,000	***P*** ** < 0.001**
Primary Language									***P*** ** < 0.001**
English	518	68.3%	197	70.1%	100	46.9%	221	83.7%	
Spanish	216	28.5%	76	27.0%	105	49.3%	35	13.3%	
Other	22	2.9%	8	2.8%	7	3.3%	7	2.7%	
English-speaking proficiency (self-assessed)									***P*** ** < 0.001**
Well/Very well	586	77.3%	225	80.1%	124	58.2%	237	89.8%	
Not well/Not at all	168	22.2%	54	19.2%	89	41.8%	25	9.5%	
Veteran	26	3.4%	8	2.8%	7	3.3%	11	4.2%	*P* = 0.91
Main Insurance									***P*** ** < 0.001**
None	58	7.7%	26	9.3%	20	9.4%	12	4.5%	
Medi-Cal	351	46.3%	104	37.0%	95	44.6%	152	57.6%	
Medicare	114	15.0%	56	19.9%	19	8.9%	39	14.8%	
Private	176	23.2%	64	22.8%	65	30.5%	47	17.8%	
Other public insurance	59	7.8%	31	11.0%	14	6.6%	14	5.3%	
Physical or mental disability affecting activities of daily living	93	12.3%	34	12.1%	47	22.1%	12	4.5%	***P*** ** < 0.001**

*GED*, general education development; *IQR*, interquartile range. Bold *P*-values indicate statistical significance.

**Table 2:** Healthcare usage and medical history by housing status.

Characteristic	Housed N = 281		Unstably housed N = 213		Homeless N = 264		*P* value
	n	%	n	%	n	%	
Health and healthcare usage characteristics - chart review							
ED visits in past 12 months, median (IQR)	2 (1–3)		2 (1–3)		2 (1–5)		***P*** **=0.017**
Hospitalizations in past 12 months, median (IQR)	0 (0-0)		0 (0-0)		0 (0-0)		*P=0.062*
Disposition							***P*** **<0.001**
Hospital admission	40	14.2%	15	7.0%	20	7.6%	
Psychiatric admission	1	0.4%	0	0.0%	9	3.4%	
Home	226	80.4%	190	89.2%	216	81.8%	
Other	14	5.0%	8	3.8%	19	7.2%	
In custody	3	1.1%	3	1.4%	12	4.5%	***P*** **=0.016**
Past medical history (last 5 visits)							
Hypertension	99	35.2%	62	29.1%	83	31.4%	*P=0.335*
Diabetes	45	16.0%	41	19.2%	42	15.9%	*P=0.555*
Stroke	15	5.3%	7	3.3%	7	2.7%	*P=0.234*
Other heart disease	27	9.6%	21	9.9%	19	7.2%	*P=0.505*
COPD	17	6.0%	7	3.3%	10	3.8%	*P=0.270*
HIV	5	1.8%	3	1.4%	7	2.7%	*P=0.597*
Depression or anxiety	32	11.4%	28	13.1%	42	15.9%	*P=0.299*
Bipolar disorder	6	2.1%	6	2.8%	18	6.8%	***P*** **=0.012**
Schizophrenia	2	0.7%	4	1.9%	20	7.6%	***P*** **<0.001**
PTSD	2	0.7%	4	1.9%	8	3.0%	*P=0.133*

*IQR*, interquartile range; *COPD*, chronic obstructive pulmonary disease; *HIV*, human immunodeficiency virus; *PTSD*, post-traumatic stress disorder. Bold *P* values indicate values that are statistically significant.

**Table 3:** Social and emotional needs by housing status included in regression analysis.

Characteristic	Housed N = 281		Unstably housed N = 213		Homeless N = 264		*P* value
	n	%	n	%	n	%	
Health and social needs characteristics - survey responses							
Unable to afford food in past 12 months	27	9.6%	58	27.2%	102	38.6%	***P*** ** < 0.001**
Unable to afford clothing in past 12 months	19	6.8%	43	20.2%	81	30.7%	***P*** ** < 0.001**
Unable to afford medicine or healthcare in past 12 months	28	10.0%	53	24.9%	99	37.5%	***P*** ** < 0.001**
Unable to afford a telephone in past 12 months	22	7.8%	45	21.1%	80	30.3%	***P*** ** < 0.001**
Utilities threatened to be shut off in past 12 months	22	7.8%	49	23.0%	78	29.5%	***P*** ** < 0.001**
Unable to afford childcare in past 12 months	9	3.2%	14	6.6%	26	9.8%	***P*** ** = 0.03**
Transportation barriers to medical care in past 12 months	33	11.7%	67	31.5%	111	42.0%	***P*** ** < 0.001**
Transportation barriers to non-medical appointments in past 12 months	33	11.7%	72	33.8%	122	46.2%	***P*** ** < 0.001**
Social and emotional health							
See or speak to people close to you less than twice per week	76	27.0%	96	45.1%	125	47.3%	***P*** ** < 0.001**
Feel stress “quite a bit” or “very much” of the time in the past 12 months	62	22.1%	81	38.0%	157	59.5%	***P*** ** < 0.001**
Incarcerated for 2 or more nights in past 12 months	14	5.0%	12	5.6%	49	18.6%	***P*** ** < 0.001**
Emotional and physical abuse							
Experienced physical abuse in the past 12 months	21	7.5%	32	15.0%	69	26.1%	***P*** ** < 0.001**
Talked down to or insulted in the past 12 months	61	21.7%	72	33.8%	131	49.6%	***P*** ** < 0.001**
Have been threatened in the past 12 months	16	5.7%	29	13.6%	69	26.1%	***P*** ** < 0.001**
Substance use history*							
Unhealthy alcohol use	92	32.7%	87	40.8%	117	44.3%	***P*** ** = 0.02**
Unhealthy prescription drug use	21	7.5%	28	13.1%	53	20.1%	***P*** ** < 0.001**
Unhealthy illegal drug use	30	10.7%	38	17.8%	81	30.7%	***P*** ** < 0.001**

* Unhealthy substance use determined using National Institute on Drug Abuse Single-Item Screening Question.
