# Supplementary material for: Association of Social Needs and Housing Status Among Urban Emergency Department Patients
Source: West J Emerg Med. 2022 Oct 28;23(6):802–10. doi: 10.5811/westjem.2022.8.55705 (PMC9683759; doi:10.5811/westjem.2022.8.55705)
Supplement: Supplementary file 1 [file wjem-23-802-s001.pdf]

HOUSED

Codebook

Data Dictionary Codebook

05/17/2021 3:28pm

#	Variable / Field Name	Field Label<br>***Field Note***	Field Attributes (Field Type, Validation, Choices, Calculations, etc.)
Instrument: **Approach** (approach)			
1	record_id	Record ID	text
2	consent_pro	Who is providing verbal consent?	radio, Required<br>1 Leah Fraimow-Wong<br>2 Daniel Haro<br>4 Matthew Lennon<br>5 Senta Wiederholt<br>6 Gray Kelsey<br>3 Other
3	other_consent_pro<br><br>Show the field ONLY if:<br>[consent_pro] = '3'	If "Other," please record your name, first and last.	text, Required
4	start_lang	English or Spanish approach script?	radio<br>1 English<br>2 Spanish

5	start_text<br><br>Show the field ONLY if:<br>[start_lang] = '1'	Hi, are you Mr./Ms. (Last Name)?<br><br>I'm (Name), and I'm a research assistant here in the emergency room. We're doing a survey with people here today about housing and other social needs. It takes about 10 to 15 minutes to complete. Can I tell you more about it?<br><br><br>IF YES:<br>Thanks for your interest! This is a survey that can be done by yourself or with me. It includes some sensitive questions about you and your experiences with housing and social services, and the information you give will be combined with other patients' answers to help us better understand this community's housing concerns.<br><br>If you participate, we'll also look at your medical records for information important to the study. No one but our researchers will see your information unless required by law.<br><br>If you feel uncomfortable at any point, you can decide to skip a question or stop doing the survey. There's no cost to you, and you won't be paid. There's no direct benefit to you. Your participation is voluntary; you can choose not to participate. Your choice will not change the care you receive. Do you have any questions?<br><br>Is it ok to continue?<br><br><br>IF PATIENT AGREES:<br>Great! Because we're asking you personal questions and looking at your health information, we have to ask for your signed permission. This document explains what information we can see and who can see it. If you are ok with this, I will have you initial in three locations, sign, and date.<br><br><br>EXPLAIN THE FORM.<br><br><br>Thank you! Before you start, I want to say that a few of the questions may be repetitive. This is on purpose and should not take much extra time!	descriptive

6	start_text_sp<br><br>Show the field ONLY i f:<br>[start_lang] = '2'	Hola, eres el Señor/Señora (Apellido)?<br><br>Soy (Nombre), y soy asistente de investigación aquí en la sala de emergencias. Estamos haciendo una encuesta con personas aquí hoy sobre vivienda y otras necesidades sociales. Tarda de 10 a 15 minutos en completarse. ¿Puedo contarte más sobre eso?<br><br><br>IF YES:<br>¡Gracias por tu interés! Esta es una encuesta que puedes hacer usted mismo o conmigo. Incluye algunas preguntas delicadas sobre usted y sus experiencias con la vivienda y los servicios sociales, y la información que tu brinde se combinará con las respuestas de otros pacientes para ayudarnos a comprender mejor las inquietudes sobre la vivienda de esta comunidad.<br><br>Si participa, también revisaremos sus registros médicos para obtener información importante para el estudio. Nadie más que nuestros investigadores verán su información a menos que lo requiera la ley.<br><br>Si en cualquier momento no se siente a gusto, puedes decidir usted saltarse una pregunta o dejar de hacer la encuesta. No hay costo para usted, y no recibirá ningún pago. No hay beneficio directo para ti. Tu participación es voluntaria; puedes elegir no participar. Su elección no cambiará la atención que recibe. ¿Tiene usted alguna pregunta?<br><br>¿Está bien continuar?<br><br><br>IF PATIENT AGREES:<br>¡Excelente! Porque le estamos haciendo preguntas personales y mirando su información de salud, debemos solicitar su permiso firmado. Este documento explica qué información podemos ver y quién puede verla. Si estás de acuerdo con esto, te pediré que inicies en tres lugares, firma y fecha.<br><br>EXPLAIN THE FORM.<br><br><br>¡Gracias! Antes de comenzar, quiero decir que algunas de las preguntas pueden ser repetitivas. ¡Esto es a propósito y no debería tomar mucho tiempo extra!	descriptive

<table>
<tr><td>7</td><td>consent</td><td>Did the patient agree to do the surveys?
By answering "Yes" to this question, you confirm that you have covered the required elements of informed consent, that the patient has had the chance to ask questions, that all their questions have been answered to their satisfaction, and that they freely agree to participate in this study. You also confirm that the patient agrees to the use, release, and disclosure of their protected health information as specified in their signed and dated authorization.</td><td>yesno, Required<table><tr><td>1</td><td>Yes</td></tr><tr><td>0</td><td>No</td></tr></table>Custom alignment: RH
Stop actions on 0</td></tr>
<tr><td>8</td><td>approach_complete</td><td>Section Header: Form Status
Complete?</td><td>dropdown<table><tr><td>0</td><td>Incomplete</td></tr><tr><td>1</td><td>Unverified</td></tr><tr><td>2</td><td>Complete</td></tr></table></td></tr>
<tr><td colspan="4">Instrument: PRAPARE 1st (prapare_1st)</td></tr>
<tr><td>9</td><td>surv_lang</td><td>Which language would you like to take the survey in?</td><td>radio, Required<table><tr><td>1</td><td>English</td></tr><tr><td>2</td><td>Spanish</td></tr></table></td></tr>
<tr><td>10</td><td>pra_start
Show the field ONLY i f:
[surv_lang] = '1'</td><td>Section Header: Personal Characteristics/ Características Personales
Please click the "Now" button on the right to record the current time.</td><td>text (datetime_seconds_mdy), Required
Custom alignment: RH</td></tr>
<tr><td>11</td><td>pra_start_sp
Show the field ONLY i f:
[surv_lang] = '2'</td><td>Por favor haga clic en el botón "Now" a la derecha para registrar la hora actual.</td><td>text (datetime_seconds_mdy), Required
Custom alignment: RH</td></tr>
<tr><td>12</td><td>race
Show the field ONLY i f:
[surv_lang] = '1'</td><td>What is your race or ethnicity? Select all that apply.</td><td>checkbox, Required<table><tr><td>11</td><td>race___11</td><td>Black/African American</td></tr><tr><td>12</td><td>race___12</td><td>White</td></tr><tr><td>13</td><td>race___13</td><td>Middle Eastern/North African</td></tr><tr><td>14</td><td>race___14</td><td>Hispanic, Latino or Spanish</td></tr><tr><td>15</td><td>race___15</td><td>Asian</td></tr><tr><td>16</td><td>race___16</td><td>Native Hawaiian</td></tr><tr><td>17</td><td>race___17</td><td>Pacific Islander</td></tr><tr><td>18</td><td>race___18</td><td>American Indian/Alaskan Native</td></tr><tr><td>19</td><td>race___19</td><td>Other</td></tr><tr><td>99</td><td>race___99</td><td>I choose not to answer this question</td></tr></table></td></tr>
</table>

13	race_sp<br><br>Show the field ONLY i f:<br>[surv_lang] = '2'	¿Cuál es su raza o etnia? Marque todo lo que sea aplicable.	checkbox, Required<br>11 race_sp___11 Negro/ Afro Americano<br>12 race_sp___12 Blanco<br>13 race_sp___13 De Medio Oriente/ Norteafricano<br>14 race_sp___14 Hispano, latino o de origen español<br>15 race_sp___15 Asiático<br>16 race_sp___16 Nativo de Hawaii<br>17 race_sp___17 De las Islas del Pacifico<br>18 race_sp___18 Indio de los Estados Unidos/ Nativo de Alaska<br>19 race_sp___19 Otro<br>99 race_sp___99 Prefiero no responder a esta pregunta
14	race_oth<br><br>Show the field ONLY i f:<br>[race(19)] = '1'	Please describe "Other."<br>*Please enter 99 if you choose not to answer this question.*	text, Required
15	race_oth_sp<br><br>Show the field ONLY i f:<br>[race_sp(19)] = '1'	Por favor describe "Otro."<br>*Por favor, escribe 99 si decide no responder a esta pregunta.*	text, Required
16	farm_work<br><br>Show the field ONLY i f:<br>[surv_lang] = '1'	At any point during the past 2 years, has seasonal or migrant farm work been your or your family's main source of income?	radio, Required<br>1 Yes<br>0 No<br>99 I choose not to answer this question
17	farm_work_sp<br><br>Show the field ONLY i f:<br>[surv_lang] = '2'	En cualquier momento en los últimos 2 años, ¿el trabajo agrícola ha sido el ingreso principal de su familia?	radio, Required<br>1 Sí<br>0 No<br>99 Prefiero no responder a esta pregunta
18	vet<br><br>Show the field ONLY i f:<br>[surv_lang] = '1'	Have you been discharged from the armed forces of the United States?	radio, Required<br>1 Yes<br>0 No<br>99 I choose not to answer this question

19	vet_sp<br><br>Show the field ONLY i f:<br>[surv_lang] = '2'	¿Ha servido en las fuerzas Armadas de los Estados Unidos?	radio, Required<br>1 Sí<br>0 No<br>99 Prefiero no responder a esta pregunta
20	lang<br><br>Show the field ONLY i f:<br>[surv_lang] = '1'	What language are you most comfortable speaking?	radio, Required<br>1 English<br>2 Spanish<br>3 Language other than English or Spanish<br>99 I choose not to answer this question
21	lang_sp<br><br>Show the field ONLY i f:<br>[surv_lang] = '2'	¿Con cuál idioma se siente más cómodo hablando?	radio<br>1 Inglés<br>2 Español<br>3 Idioma aparte del Inglés o Español<br>99 Prefiero no responder a esta pregunta
22	lang_oth<br><br>Show the field ONLY i f:<br>[lang] = '3'	Please describe "Other."<br>*Please enter 99 if you choose not to answer this question.*	text, Required<br>Custom alignment: RH
23	lang_oth_sp<br><br>Show the field ONLY i f:<br>[lang_sp] = '3'	Por favor describe "Otro."<br>*Por favor, escribe 99 si decide no responder a esta pregunta.*	text, Required
24	fam_home<br><br>Show the field ONLY i f:<br>[surv_lang] = '1'	Section Header: *Family & Home/ Familia y Hogar*<br>How many family members, including yourself, do you currently live with?<br>*Please enter 99 if you choose not to answer this question.*	text (integer), Required<br>Custom alignment: RH
25	fam_home_sp<br><br>Show the field ONLY i f:<br>[surv_lang] = '2'	¿Cuántos miembros de su familia principal viven con usted? (incluido usted mismo)<br>*Por favor, escribe 99 si decide no responder a esta pregunta.*	text (integer), Required<br>Custom alignment: RH
26	house_tdy_pra<br><br>Show the field ONLY i f:<br>[surv_lang] = '1'	What is your housing situation today?	radio, Required<br>1 I have housing<br>2 I do not have housing (staying with others, in a hotel, in a shelter, living outside on the street, on a beach, in a car, or in a park)<br>99 I choose not to answer this question

27	house_tdy_pra_sp<br>Show the field ONLY if:<br>[surv_lang] = '2'	¿Cuál es su situación actualmente de su vivienda?	radio<br>1 Tengo vivienda<br>2 No tengo vivienda (viviendo con otros, en un hotel, en un albergue, viviendo en la calle, en una playa, en un carro, o en un parque)<br>99 Prefiero no responder a esta pregunta
28	house_worried<br>Show the field ONLY if:<br>[surv_lang] = '1'	Are you worried about losing your housing?	radio, Required<br>1 Yes<br>0 No<br>2 N/A (not applicable)<br>99 I choose not to answer this question
29	house_worried_sp<br>Show the field ONLY if:<br>[surv_lang] = '2'	¿Le preocupa que pudiera perder su vivienda?	radio, Required<br>1 Sí<br>0 No<br>2 N/A (no aplica)<br>99 Prefiero no responder a esta pregunta
30	street<br>Show the field ONLY if:<br>[surv_lang] = '1'	What address do you live at?<br>*Street number and name. Please enter 99 if N/A or if you choose not to answer this question.*	text, Required, Identifier
31	street_sp<br>Show the field ONLY if:<br>[surv_lang] = '2'	¿Cuál es su dirección de hogar?<br>*Número y nombre de la calle. Por favor, escribe 99 si no corresponde o si decide no responder a esta pregunta.*	text, Required, Identifier
32	city_state<br>Show the field ONLY if:<br>[surv_lang] = '1'	City and state<br>*Please enter 99 if you choose not to answer this question.*	text, Required, Identifier
33	city_state_sp<br>Show the field ONLY if:<br>[surv_lang] = '2'	Ciudad y estado<br>*Por favor, escribe 99 si decide no responder a esta pregunta.*	text, Required, Identifier
34	zip_code<br>Show the field ONLY if:<br>[surv_lang] = '1'	Zip Code<br>*Please enter 99999 if N/A or if you choose not to answer this question.*	text (zipcode), Required, Identifier

35	zip_code_sp<br><br>Show the field ONLY if:<br>[surv_lang] = '2'	Código postal<br>*Por favor, escribe 99 si no corresponde o si decide no responder a esta pregunta.*	text (zipcode), Required, Identifier
36	highest_edu<br><br>Show the field ONLY if:<br>[surv_lang] = '1'	Section Header: *Money & Resources/ Dinero y Recursos*<br>What is the highest level of school that you have finished?	radio, Required<br>1 Less than a high school degree<br>2 High school diploma or GED<br>3 More than high school<br>99 I choose not to answer this question
37	highest_edu_sp<br><br>Show the field ONLY if:<br>[surv_lang] = '2'	¿Cuál es el nivel escolar más alto que ha completado?	radio, Required<br>1 Escuela primaria<br>2 Preparatoria<br>3 Colegio, Universidad, o colegio técnico<br>99 Prefiero no responder a esta pregunta
38	work_now<br><br>Show the field ONLY if:<br>[surv_lang] = '1'	What is your current work situation?	radio, Required<br>1 Unemployed and seeking work<br>2 Part-time or temporary work<br>3 Full-time work<br>4 Otherwise unemployed but not seeking work (ex. student, retired, disabled, unpaid primary caregiver)<br>99 I choose not to answer this question
39	work_now_sp<br><br>Show the field ONLY if:<br>[surv_lang] = '2'	¿Cuál es su situación laboral actualmente?	radio, Required<br>1 Desempleado y buscando empleo<br>2 Trabajo tiempo parcial o temporal (no agrícola)<br>3 Trabajo tiempo completo<br>4 Desempleado (ej. estudiante, jubilado, incapacitado, cuidador principal no remunerado)<br>99 Prefiero no responder a esta pregunta
40	work_oth<br><br>Show the field ONLY if:<br>[work_now] = '4'	Please describe above answer.<br>*Please enter 99 if you choose not to answer this question.*	text, Required

41	work_oth_sp<br>Show the field ONLY if:<br>[work_now_sp] = '4'	Por favor, describe la respuesta anterior.<br>*Por favor, escribe 99 si decide no responder a esta pregunta.*	text, Required
42	insurance<br>Show the field ONLY if:<br>[surv_lang] = '1'	What is your main insurance?	radio, Required<br>1 None/ uninsured<br>2 Medicaid (Medi-Cal)<br>3 CHIP Medicaid<br>4 Medicare<br>5 Other public insurance (not CHIP)<br>6 Other public insurance (CHIP)<br>7 Private insurance<br>99 I choose not to answer this question
43	insurance_sp<br>Show the field ONLY if:<br>[surv_lang] = '2'	¿Cuál es su aseguranza médica?	radio, Required<br>1 Ninguno/ no asegurado<br>2 Medicaid (Medi-Cal)<br>3 CHIP Medicaid<br>4 Medicare<br>5 Otra aseguranza pública (No CHIP)<br>6 Otra aseguranza pública (CHIP)<br>7 Aseguranza privada<br>99 Prefiero no responder a esta pregunta
44	income<br>Show the field ONLY if:<br>[surv_lang] = '1'	During the past year, what was the total combined income for you and your family members you live with?<br>*This information will help us determine if you are eligible for any benefits. Please enter 99 if you choose not to answer this question.*	text (number), Required
45	income_sp<br>Show the field ONLY if:<br>[surv_lang] = '2'	¿Cuál fue el ingreso de su familia el año pasado?<br>*Esta información nos ayudará a determiner si usted califica para algún beneficio. Por favor, escribe 99 si decide no responder a esta pregunta.*	text (number), Required
46	needs_food	Section Header: *In the past year, have you or any family members you live with been unable to get any of the following when it was really needed? Select all that apply. Actualmente o el año pasado, ¿usted o alguna persona en su hogar tuvieron que privarse de algo que realmente se necesitaba? Marque todo lo que sea aplicable.*<br>Food/ Alimentos	radio (Matrix), Required<br>1 Yes/ Sí<br>0 No<br>99 I choose not to answer this question/ Prefiero no responder a esta pregunta

47	needs_utilities	Utilities/ Servicios públicos	radio (Matrix), Required<br>1 Yes/ Sí<br>0 No<br>99 I choose not to answer this question/ Prefiero no responder a esta pregunta
48	needs_med	Medicine or any healthcare (medical, dental, mental health, vision)/ Medicina o cualquier cuidado de salud (medico, dental, salud mental, vision)	radio (Matrix), Required<br>1 Yes/ Sí<br>0 No<br>99 I choose not to answer this question/ Prefiero no responder a esta pregunta
49	needs_phone	Phone/ Teléfono	radio (Matrix), Required<br>1 Yes/ Sí<br>0 No<br>99 I choose not to answer this question/ Prefiero no responder a esta pregunta
50	needs_clothing	Clothing/ Ropa	radio (Matrix), Required<br>1 Yes/ Sí<br>0 No<br>99 I choose not to answer this question/ Prefiero no responder a esta pregunta
51	needs_child	Child care/ Cuidado infantil	radio (Matrix), Required<br>1 Yes/ Sí<br>0 No<br>99 I choose not to answer this question/ Prefiero no responder a esta pregunta
52	needs_oth	Other/ Otro	radio (Matrix), Required<br>1 Yes/ Sí<br>0 No<br>99 I choose not to answer this question/ Prefiero no responder a esta pregunta
53	needs_oth_pra<br>Show the field ONLY if:<br>[needs_oth] = '1' and [surv_lang] = '1'	Please describe "Other."<br>*Please enter 99 if you choose not to answer this question.*	text, Required

54	needs_oth_pra_sp<br><br>Show the field ONLY if:<br>[needs_oth] = '1' and [surv_lang] = '2'	Por favor describe "Otro."<br>*Por favor, escribe 99 si decide no responder a esta pregunta.*	text, Required
55	transport_pra<br><br>Show the field ONLY if:<br>[surv_lang] = '1'	Has lack of transportation kept you from medical appointments, meetings, work, or from getting things needed for daily living? Select all that apply.	checkbox, Required<br>1 | transport_pra___1 | Yes, it has kept me from medical appointments or from getting my medications<br>2 | transport_pra___2 | Yes, it has kept me from non-medical meetings, appointments, work, or from getting things I need<br>0 | transport_pra___0 | No<br>99 | transport_pra___99 | I choose not to answer this question
56	transport_pra_sp<br><br>Show the field ONLY if:<br>[surv_lang] = '2'	¿La falta de transportación le ha impedido ir a citas médicas, a reuniones, al trabajo, o conseguir cosas necesarias para la vida diaria? Marque todas las que aplican.	checkbox, Required<br>1 | transport_pra_sp___1 | Sí, me ha impedido ir a citas médicas o a recoger mis medicamentos<br>2 | transport_pra_sp___2 | Sí, me ha impedido ir a reunions o citas no médicas, al trabajo, o conseguir cosas que necesito<br>0 | transport_pra_sp___0 | No<br>99 | transport_pra_sp___99 | Prefiero no responder a esta pregunta

57	social<br><br>Show the field ONLY i f:<br>[surv_lang] = '1'	Section Header: *Social & Emotional Health/ Salud Social y Emocional*<br><br>How often do you see or talk to people that you care about and feel close to? (For example: talking to friends on the phone, visiting friends or family, going to church or club meetings)	radio, Required<br>1 Less than once a week<br>2 1 or 2 times a week<br>3 3 to 5 times a week<br>4 More than 5 times a week<br>99 I choose not to answer this question
58	social_sp<br><br>Show the field ONLY i f:<br>[surv_lang] = '2'	¿Con qué frecuencia convive o conversa con personas por las que se preocupa y son cercanas a usted? (Por ejemplo: conversar con amigos por teléfono, visitar a amigos o familiares, asistir a la iglesia o reuniones)	radio, Required<br>1 Menos de una vez por semana<br>2 1 o 2 veces por semana<br>3 De 3 a 5 veces por semana<br>4 Más de 5 veces por semana<br>99 Prefiero no responder a esta pregunta
59	stress<br><br>Show the field ONLY i f:<br>[surv_lang] = '1'	Stress is when someone feels tense, nervous, anxious, or can't sleep at night because their mind is troubled. How stressed are you?	radio, Required<br>1 Not at all<br>2 A little bit<br>3 Somewhat<br>4 Quite a bit<br>5 Very much<br>99 I choose not to answer this question
60	stress_sp<br><br>Show the field ONLY i f:<br>[surv_lang] = '2'	Estrés es cuando alguien se siente tenso, nervioso o no puede dormir en la noche porque su mente está preocupada. ¿Usted se siente estresado?	radio, Required<br>1 Para nada<br>2 Un poquito<br>3 Algunas veces<br>4 Bastante<br>5 Mucho<br>99 Prefiero no responder a esta pregunta
61	jail<br><br>Show the field ONLY i f:<br>[surv_lang] = '1'	In the past year, have you spent more than 2 nights in a row in a jail, prison, detention center, or juvenile correction facility?	radio, Required<br>1 Yes<br>0 No<br>99 I choose not to answer this question
62	jail_sp<br><br>Show the field ONLY i f:<br>[surv_lang] = '2'	En el último año, ¿ha pasado más de 2 noches seguidas en una cárcel, una prisión, un centro de detención, o en un centro correccional juvenil?	radio, Required<br>1 Sí<br>0 No<br>99 Prefiero no responder a esta pregunta

63	pra_end_ahc_start<br>Show the field ONLY i f:<br>[surv_lang] = '1'	Please click the "Now" button on the right to record the current time.	text (datetime_seconds_mdy), Required<br>Custom alignment: RH
64	pra_end_ahc_start_sp<br>Show the field ONLY i f:<br>[surv_lang] = '2'	Por favor haga clic en el botón "Now" a la derecha para registrar la hora actual.	text (datetime_seconds_mdy), Required<br>Custom alignment: RH
65	house_tdy_ahc<br>Show the field ONLY i f:<br>[surv_lang] = '1'	Section Header: *Housing Instability & Food Insecurity/ Inestabilidad de vivienda & Inseguridad alimentaria*<br>What is your housing situation today?	radio, Required<br>1 — I have a steady place to live.<br>2 — I have housing today, but I am worried about losing it in the future.<br>3 — I do not have a steady place to live (I am temporarily staying with others, in a hotel, in a shelter, living outside on the street, on a beach, in a car, abandoned building, bus or train station, or in a park).<br>99 — I choose not to answer this question.
66	house_tdy_ahc_sp<br>Show the field ONLY i f:<br>[surv_lang] = '2'	¿Cuál es su situación de vivienda hoy?	radio, Required<br>1 — Tengo un lugar estable para vivir<br>2 — Tengo vivienda hoy, pero me preocupa perderla en el futuro<br>3 — No tengo un lugar estable para vivir (Me estoy quedando temporalmente con otros, en un hotel, en un refugio, viviendo afuera en la calle, en una playa, en un automóvil, en un edificio abandonado, en una estación de autobuses o de tren, o en un parque)<br>99 — Prefiero no responder a esta pregunta

67	house_problems<br><br>Show the field ONLY if:<br>[surv_lang] = '1'	Think about the place you live. Do you have problems with any of the following? Select all that apply.	checkbox, Required<br>1 house_problems___1 Pests such as bugs, ants, or mice<br>2 house_problems___2 Mold<br>3 house_problems___3 Lead paint or pipes<br>4 house_problems___4 Lack of heat<br>5 house_problems___5 Oven or stove not working<br>6 house_problems___6 Smoke detectors missing or not working<br>7 house_problems___7 Water leaks<br>8 house_problems___8 None of the above<br>99 house_problems___99 I choose not to answer this question

68	house_problems_sp<br>Show the field ONLY i f:<br>[surv_lang] = '2'	Piensa en el lugar donde vives. ¿Tiene problemas con alguno de los siguientes? Marque todo lo que corresponda.	checkbox, Required<br>1 | house_problems_sp___1 | Plagas como insectos, hormigas o ratones<br>2 | house_problems_sp___2 | Molde<br>3 | house_problems_sp___3 | Pintura o tubos con plomo<br>4 | house_problems_sp___4 | Falta de calentón<br>5 | house_problems_sp___5 | Horno o estufa que no funciona<br>6 | house_problems_sp___6 | Detectores de humo que faltan o no funcionan<br>7 | house_problems_sp___7 | Fugas de agua<br>8 | house_problems_sp___8 | Ninguna de las respuestas<br>99 | house_problems_sp___99 | Prefiero no responder a esta pregunta
69	ahc_food_text<br>Show the field ONLY i f:<br>[surv_lang] = '1'	Some people have made the following statements about their food situation. Please answer whether the statements were OFTEN, SOMETIMES, or NEVER true for you and your household in the last 12 months.	descriptive
70	ahc_food_text_sp<br>Show the field ONLY i f:<br>[surv_lang] = '2'	Algunas personas han hecho las siguientes declaraciones sobre su situación alimentaria. Por favor, responda si las declaraciones fueron FRECUENTES, A VECES o NUNCA verdad para usted y su hogar en los últimos 12 meses.	descriptive
71	food_worried<br>Show the field ONLY i f:<br>[surv_lang] = '1'	Within the past 12 months, you worried that your food would run out before you got money to buy more.	radio, Required<br>1 | Often true<br>2 | Sometimes true<br>3 | Never true<br>99 | I choose not to answer this question

72	food_worried_sp<br><br>Show the field ONLY if:<br>[surv_lang] = '2'	En los últimos 12 meses, le preocupaba que su comida se acabado antes de que tenga dinero para comprar más.	radio, Required<br>1 Con frecuencia<br>2 A veces<br>3 Nunca<br>99 Prefiero no responder a esta pregunta
73	food_ran_out<br><br>Show the field ONLY if:<br>[surv_lang] = '1'	Within the past 12 months, the food you bought just didn't last and you didn't have money to get more.	radio, Required<br>1 Often true<br>2 Sometimes true<br>3 Never true<br>99 I choose not to answer this question
74	food_ran_out_sp<br><br>Show the field ONLY if:<br>[surv_lang] = '2'	En los últimos 12 meses, la comida que compró simplemente no duró y no tenía dinero para obtener más.	radio, Required<br>1 Con frecuencia<br>2 A veces<br>3 Nunca<br>99 Prefiero no responder a esta pregunta
75	transport_ahc<br><br>Show the field ONLY if:<br>[surv_lang] = '1'	Section Header: *Transportation Needs, Utility Needs & Safety/ Necesidades de transporte, Necesidades de utilidad & La seguridad*<br><br>In the past 12 months, has lack of transportation kept you from medical appointments, meetings, work or from getting things needed for daily living?	radio, Required<br>1 Yes<br>0 No<br>99 I choose not to answer this question
76	transport_ahc_sp<br><br>Show the field ONLY if:<br>[surv_lang] = '2'	En los últimos 12 meses, ¿la falta de transporte lo mantuvo alejado de citas médicas, reuniones, trabajo o de obtener las cosas necesarias para la vida diaria?	radio, Required<br>1 Sí<br>0 No<br>99 Prefiero no responder a esta pregunta
77	utilities_threat<br><br>Show the field ONLY if:<br>[surv_lang] = '1'	In the past 12 months, has the electric, gas, oil, or water company threatened to shut off services in your home?	radio, Required<br>1 Yes<br>0 No<br>2 Already shut off<br>99 I choose not to answer this question
78	utilities_threat_sp<br><br>Show the field ONLY if:<br>[surv_lang] = '2'	En los últimos 12 meses, ¿la compañía de electricidad, gas, petróleo o agua ha amenazado con cerrar los servicios en su hogar?	radio, Required<br>1 Sí<br>0 No<br>2 Ya apagó<br>99 Prefiero no responder a esta pregunta

79	ahc_safety_text<br>Show the field ONLY i f:<br>[surv_lang] = '1'	Because violence and abuse happen to a lot of people and affects their health, we are asking the following questions.	descriptive
80	ahc_safety_text_sp<br>Show the field ONLY i f:<br>[surv_lang] = '2'	Debido a que la violencia y el abuso le ocurren a mucha gente y afectan su salud, nos hacemos las siguientes preguntas.	descriptive
81	abuse_phys<br>Show the field ONLY i f:<br>[surv_lang] = '1'	How often does anyone, including family, physically hurt you?	radio, Required<br>1 Never<br>2 Rarely<br>3 Sometimes<br>4 Fairly often<br>5 Frequently<br>99 I choose not to answer this question
82	abuse_phys_sp<br>Show the field ONLY i f:<br>[surv_lang] = '2'	¿Con qué frecuencia alguien, incluida la familia, lo lastima físicamente?	radio, Required<br>1 Nunca<br>2 Raramente<br>3 A veces<br>4 Bastante<br>5 Frecuentemente<br>99 Prefiero no responder a esta pregunta
83	abuse_belittle<br>Show the field ONLY i f:<br>[surv_lang] = '1'	How often does anyone, including family, insult or talk down to you?	radio, Required<br>1 Never<br>2 Rarely<br>3 Sometimes<br>4 Fairly often<br>5 Frequently<br>99 I choose not to answer this question
84	abuse_belittle_sp<br>Show the field ONLY i f:<br>[surv_lang] = '2'	¿Con qué frecuencia alguien, incluida la familia, le insulta o le critica?	radio, Required<br>1 Nunca<br>2 Raramente<br>3 A veces<br>4 Bastante<br>5 Frecuentemente<br>99 Prefiero no responder a esta pregunta

85	abuse_threats<br><br>Show the field ONLY if:<br>[surv_lang] = '1'	How often does anyone, including family, threaten you with harm?	radio, Required<br>1 Never<br>2 Rarely<br>3 Sometimes<br>4 Fairly often<br>5 Frequently<br>99 I choose not to answer this question
86	abuse_threats_sp<br><br>Show the field ONLY if:<br>[surv_lang] = '2'	¿Con qué frecuencia alguien, incluida la familia, lo amenaza con daño?	radio, Required<br>1 Nunca<br>2 Raramente<br>3 A veces<br>4 Bastante<br>5 Frecuentemente<br>99 Prefiero no responder a esta pregunta
87	abuse_curse<br><br>Show the field ONLY if:<br>[surv_lang] = '1'	How often does anyone, including family, scream or curse at you?	radio, Required<br>1 Never<br>2 Rarely<br>3 Sometimes<br>4 Fairly often<br>5 Frequently<br>99 I choose not to answer this question
88	abuse_curse_sp<br><br>Show the field ONLY if:<br>[surv_lang] = '2'	¿Con qué frecuencia alguien, incluida la familia, grita o maldice?	radio, Required<br>1 Nunca<br>2 Raramente<br>3 A veces<br>4 Bastante<br>5 Frecuentemente<br>99 Prefiero no responder a esta pregunta
89	decision_making<br><br>Show the field ONLY if:<br>[surv_lang] = '1'	Section Header: *Disabilities & Substance Use/ Discapacidades & Uso de sustancias*<br>Because of a physical, mental, or emotional condition, do you have serious difficulty concentrating, remembering, or making decisions?	radio, Required<br>1 Yes<br>0 No<br>99 I choose not to answer this question

90	decision_making_sp<br><br>Show the field ONLY i f:<br>[surv_lang] = '2'	Debido a una condición física, mental o emocional, ¿tiene serias dificultades para concentrarse, recordar o tomar decisiones?	radio, Required<br>1 Sí<br>0 No<br>99 Prefiero no responder a esta pregunta
91	daily_life<br><br>Show the field ONLY i f:<br>[surv_lang] = '1'	Because of a physical, mental, or emotional condition, do you have difficulty doing errands alone such as visiting a doctor's office or shopping?	radio, Required<br>1 Yes<br>0 No<br>99 I choose not to answer this question
92	daily_life_sp<br><br>Show the field ONLY i f:<br>[surv_lang] = '2'	Debido a una condición física, mental o emocional, ¿tiene dificultades para hacer mandados solo, como visitar el consultorio de un médico o ir de compras?	radio, Required<br>1 Sí<br>0 No<br>99 Prefiero no responder a esta pregunta
93	ahc_subs_use_text<br><br>Show the field ONLY i f:<br>[surv_lang] = '1'	The next questions relate to your experience with alcohol, cigarettes, and other drugs. Some of the substances are prescribed by a doctor (like pain medications), but only count those if you have taken them for reasons or in doses other than prescribed. One question is about illicit or illegal drug use, but we only ask in order to identify community services that may be available to help you.	descriptive
94	ahc_subs_use_text_sp<br><br>Show the field ONLY i f:<br>[surv_lang] = '2'	Las siguientes preguntas se relacionan con su experiencia con el alcohol, cigarrillos y otras drogas. Algunas de las sustancias son recetadas por un médico (como medicamentos para el dolor), pero solo cuéntelas si las ha tomado por razones o en dosis distintas a las recetadas. Una pregunta es acerca del uso ilícito o ilegal de drogas, pero solo preguntamos para identificar los servicios comunitarios que pueden estar disponibles para ayudarlo.	descriptive
95	etoh<br><br>Show the field ONLY i f:<br>[surv_lang] = '1'	How many times in the past 12 months have you had 5 or more drinks in a day (males) or 4 or more drinks in a day (females)?<br>*One drink is 12 ounces of beer, 5 ounces of wine, or 1.5 ounces of 80-proof spirits.*	radio, Required<br>1 Never<br>2 Once or twice<br>3 Monthly<br>4 Weekly<br>5 Daily or almost daily<br>99 I choose not to answer this question

96	etoh_sp<br><br>Show the field ONLY i f:<br>[surv_lang] = '2'	¿Cuántas veces en los últimos 12 meses ha bebido 5 o más bebidas en un día (hombres) o 4 o más bebidas en un día (mujeres)?<br>*Una bebida es 12 onzas de cerveza, 5 onzas de vino o 1.5 onzas de espíritus de 80 pruebas.*	radio, Required<br>1 Nunca<br>2 Una o dos veces<br>3 Mensual<br>4 Semanal<br>5 Diariamente o casi a diario<br>99 Prefiero no responder a esta pregunta
97	drugs_rx<br><br>Show the field ONLY i f:<br>[surv_lang] = '1'	How many times in the past year have you used prescription drugs for non-medical reasons?	radio, Required<br>1 Never<br>2 Once or twice<br>3 Monthly<br>4 Weekly<br>5 Daily or almost daily<br>99 I choose not to answer this question
98	drugs_rx_sp<br><br>Show the field ONLY i f:<br>[surv_lang] = '2'	¿Cuántas veces en el último año ha usado medicamentos recetados por razones no médicas?	radio, Required<br>1 Nunca<br>2 Una o dos veces<br>3 Mensual<br>4 Semanal<br>5 Diariamente o casi a diario<br>99 Prefiero no responder a esta pregunta
99	drugs_illegal<br><br>Show the field ONLY i f:<br>[surv_lang] = '1'	How many times in the past year have you used illegal drugs?	radio, Required<br>1 Never<br>2 Once or twice<br>3 Monthly<br>4 Weekly<br>5 Daily or almost daily<br>99 I choose not to answer this question

100	drugs_illegal_sp<br>Show the field ONLY if:<br>[surv_lang] = '2'	¿Cuántas veces en el último año ha usado drogas ilegales?	radio, Required<br>1 Nunca<br>2 Una o dos veces<br>3 Mensual<br>4 Semanal<br>5 Diariamente o casi a diario<br>99 Prefiero no responder a esta pregunta
101	ahc_end_house_start<br>Show the field ONLY if:<br>[surv_lang] = '1'	Please click the "Now" button on the right to record the current time.	text (datetime_seconds_mdy), Required
102	ahc_end_house_start_sp<br>Show the field ONLY if:<br>[surv_lang] = '2'	Por favor haga clic en el botón "Now" a la derecha para registrar la hora actual.	text (datetime_seconds_mdy), Required
103	drugs_inj<br>Show the field ONLY if:<br>[surv_lang] = '1'	Have you ever used any drug by injection (non-medical use only)?	radio, Required<br>1 Yes<br>0 No<br>99 I choose not to answer this question
104	drugs_inj_sp<br>Show the field ONLY if:<br>[surv_lang] = '2'	¿Alguna vez ha usado algún medicamento por inyección (solo para uso no médico)?	radio, Required<br>1 Sí<br>0 No<br>99 Prefiero no responder a esta pregunta
105	drugs_inj_when<br>Show the field ONLY if:<br>[drugs_inj] = '1'	When was the last time you injected?	radio, Required<br>1 In the past 90 days<br>2 In the past year<br>3 Over a year ago<br>99 I choose not to answer this question
106	drugs_inj_when_sp<br>Show the field ONLY if:<br>[drugs_inj_sp] = '1'	¿Cuándo fue la última vez que le inyectaron a usted?	radio, Required<br>1 En los últimos 90 días<br>2 En el año pasado<br>3 Hace más de un año<br>99 Prefiero no responder a esta pregunta

107	eng_ability<br><br>Show the field ONLY if:<br>[surv_lang] = '1'	Section Header: *Final Questions - Housing Focus/ Preguntas finales - Enfoque en la vivienda*<br><br>How well do you speak English?	radio, Required<br>1 Very well<br>2 Well<br>3 Not well<br>4 Not at all<br>99 I choose not to answer this question
108	eng_ability_sp<br><br>Show the field ONLY if:<br>[surv_lang] = '2'	¿Que tan bien hablas ingles?	radio, Required<br>1 Muy bien<br>2 Bien<br>3 No muy bien<br>4 Mal<br>99 Prefiero no responder a esta pregunta
109	gender<br><br>Show the field ONLY if:<br>[surv_lang] = '1'	What is your current gender identity?	radio, Required<br>1 Female/ Woman<br>2 Male/ Man<br>3 Trans male/ Trans man<br>4 Trans female/ Trans woman<br>5 Genderqueer/ Gender non-conforming<br>6 Different identity<br>99 I choose not to answer this question
110	gender_sp<br><br>Show the field ONLY if:<br>[surv_lang] = '2'	¿Cuál es su identidad preferencia de género actual?	radio, Required<br>1 Mujer<br>2 Hombre<br>3 Hombre trans<br>4 Mujer trans<br>5 Genderqueer/ Gender no conformes<br>6 Identidad diferente<br>99 Prefiero no responder a esta pregunta

111	moves<br><br>Show the field ONLY i f:<br>[surv_lang] = '1'	How many times have you moved in the past 12 months?	radio, Required<br>0 0<br>1 1<br>2 2<br>3 3<br>4 4 or more<br>99 I choose not to answer this question
112	moves_sp<br><br>Show the field ONLY i f:<br>[surv_lang] = '2'	¿Cuántas veces se ha mudado en los últimos 12 meses?	radio, Required<br>0 0<br>1 1<br>2 2<br>3 3<br>4 4 o más<br>99 Prefiero no responder a esta pregunta
113	house_unable<br><br>Show the field ONLY i f:<br>[surv_lang] = '1'	During the last 12 months, was there a time when you or you and your family were not able to pay your mortgage, rent, or utility bills?	radio, Required<br>1 Yes<br>0 No<br>2 Unsure<br>3 I have been homeless<br>99 I choose not to answer this question
114	house_unable_sp<br><br>Show the field ONLY i f:<br>[surv_lang] = '2'	Durante los últimos 12 meses, ¿hubo un momento en que usted o usted y su familia no pudieron pagar su hipoteca, renta o utilidades?	radio, Required<br>1 Sí<br>0 No<br>2 No estoy seguro<br>3 He estado sin hogar<br>99 Prefiero no responder a esta pregunta
115	needs_move<br><br>Show the field ONLY i f:<br>[surv_lang] = '1'	During the last 12 months, did you or your children move in with other people even for a little while because you could not afford to pay your mortgage, rent, or utility bills?	radio, Required<br>1 Yes<br>0 No<br>2 Unsure<br>3 I have been homeless<br>99 I choose not to answer this question

116	needs_move_sp<br><br>Show the field ONLY if:<br>[surv_lang] = '2'	Durante los últimos 12 meses, ¿usted o sus hijos se mudaron con otras personas, incluso por un tiempo, porque no podía pagar su hipoteca, renta o utilidades?	radio, Required<br>1 Sí<br>0 No<br>2 No estoy seguro<br>3 He estado sin hogar<br>99 Prefiero no responder a esta pregunta

117	last_night<br><br>Show the field ONLY i f:<br>[surv_lang] = '1'	Where did you stay last night? Please select the one response that best describes where you stayed last night.	radio, Required<br>1 Emergency shelter, including hotel or motel voucher paid for by a social service or charitable organization<br>2 Transitional housing for homeless persons<br>3 Permanent supportive housing for formerly homeless persons<br>4 Psychiatric hospital or other psychiatric facility<br>5 Substance abuse treatment facility or other detox facility<br>6 Hospital (non-psychiatric)<br>7 Jail, prison or juvenile detention facility<br>8 Half-way or three-quarter-way home for persons with criminal offenses<br>9 Room, apartment or house that you rent<br>10 Apartment or house that you own<br>11 In a friend's or family member's room, apartment or house<br>12 Hotel or motel paid for without emergency shelter voucher<br>13 Foster care home or foster care group home<br>14 Group home or other supervised residential care facility<br>15 Place not meant for human habitation (street, car, park, etc.)<br>16 Place or a situation that is dangerous to the health or safety of any household member<br>17 Other<br>99 I choose not to answer this question

118	last_night_sp<br><br>Show the field ONLY if:<br>[surv_lang] = '2'	¿Dónde te quedaste anoche? Seleccione la respuesta que mejor describa dónde se hospedó anoche.	radio, Required<br>1 Refugio de emergencia, incluyendo bono de hotel o motel pagado por un servicio social o organización benéfica<br>2 Vivienda de transición para personas sin hogar<br>3 Vivienda de apoyo permanente para personas que anteriormente no tenían hogar<br>4 Hospital psiquiátrico u otras facilidades psiquiátricas<br>5 Centro de tratamiento de abuso de sustancias o otras instalaciones de desintoxicación<br>6 Hospital (no psiquiátrico)<br>7 Cárcel, prisión o centro de detención juvenil<br>8 Hogar de transición para personas con ofensas criminales<br>9 Habitación, apartamento o casa que renta<br>10 Apartmento o casa que usted dueño de<br>11 En la habitación, apartamento o casa de un amigo o familiar<br>12 Hotel o motel pagado sin bono de refugio de emergencia<br>13 Hogar de cuidado de crianza o hogar de grupo de cuidado de crianza (foster care)<br>14 Hogar grupal o otro centro residencial de cuidado supervisado<br>15 Lugar no destinado a la habitación humana (calle, coche, parque, etc.)<br>16 Lugar o una situación que es peligrosa para la salud o la seguridad de cualquier miembro del hogar<br>17 Otro<br>99 Prefiero no responder a esta pregunta
119	last_night_oth<br><br>Show the field ONLY if:<br>[last_night] = '17'	Please describe "Other."<br>*Please enter 99 if you choose not to answer this question.*	text, Required

120	last_night_oth_sp<br><br>Show the field ONLY if:<br>[last_night_sp] = '17'	Por favor describe "Otro."<br>*Por favor, escribe 99 si decide no responder a esta pregunta.*	text, Required
121	last_night_length<br><br>Show the field ONLY if:<br>[surv_lang] = '1'	How long have you stayed in the place you stayed last night?	radio, Required<br>1 One week or less<br>2 More than one week, but less than one month<br>3 One to three months<br>4 More than three months, but less than one year<br>5 One year or longer<br>99 I choose not to answer this question
122	last_night_length_sp<br><br>Show the field ONLY if:<br>[surv_lang] = '2'	¿Cuánto tiempo hace que te quedaste en el lugar donde te hospedaste anoche?	radio, Required<br>1 Una semana o menos<br>2 Más de una semana, pero menos de un mes<br>3 De uno a tres meses<br>4 Más de tres meses, pero menos de un año<br>5 Un año o más<br>99 Prefiero no responder a esta pregunta
123	stay_90d<br><br>Show the field ONLY if:<br>[surv_lang] = '1'	Are you able to stay in this place for more than 90 days?	radio, Required<br>1 Yes<br>0 No/ Unsure<br>2 I am currently homeless<br>99 I choose not to answer this question
124	stay_90d_sp<br><br>Show the field ONLY if:<br>[surv_lang] = '2'	¿Puedes quedarte en este lugar por más de 90 días?	radio, Required<br>1 Sí<br>0 No/ No estoy seguro<br>2 Estoy sin hogar<br>99 Prefiero no responder a esta pregunta
125	leave_reason<br><br>Show the field ONLY if:<br>[stay_90d] = '0'	Why do you need or want to leave? Please select all of the reasons why you need to leave the place you stayed last night.	checkbox, Required<br>1 leave_reason___1 Received an eviction notice<br>2 leave_reason___2 Non-payment of rent or past due rent

3	leave_reason___3	Unable to pay future rent because lost housing subsidy, job, or other income source
4	leave_reason___4	Non-payment of utilities or utility shut-off
5	leave_reason___5	Overcrowding
6	leave_reason___6	Inability to contribute to household costs
7	leave_reason___7	Housekeeping concerns (failure to maintain cleanliness of the unit)
8	leave_reason___8	Housing is or will be condemned
9	leave_reason___9	Friend or family member being evicted or threatened with eviction
10	leave_reason___10	Threat of abuse by partner, family member, or other
11	leave_reason___11	Being discharged or service is being terminated
12	leave_reason___12	Personal conflict with others
13	leave_reason___13	Other health or safety concerns
14	leave_reason___14	Other lease violation(s)
15	leave_reason___15	Other

			99 | leave_reason___99 | I choose not to answer this question
126	leave_reason_sp<br>Show the field ONLY i f:<br>[stay_90d_sp] = '0'	¿Por qué necesitas o quieres irte? Por favor, marque todos los motivos por los que debe abandonar el lugar donde se alojó anoche.	checkbox, Required<br>1 | leave_reason_sp___1 | Recibió un aviso de desalojo<br>2 | leave_reason_sp___2 | Falta de pago de renta o renta vencida<br>3 | leave_reason_sp___3 | No se puede pagar la renta futura porque se perdió el subsidio de vivienda, el trabajo o otra fuente de ingresos<br>4 | leave_reason_sp___4 | Falta de pago de las utilidades o corte de utilidades<br>5 | leave_reason_sp___5 | Superpoblació<br>6 | leave_reason_sp___6 | No poder contribuir a los costos del hogar<br>7 | leave_reason_sp___7 | Problemas de limpieza (falta de mantenimient de la limpieza de la unidad)<br>8 | leave_reason_sp___8 | La vivienda es [illegible] será declarado en ruinas<br>9 | leave_reason_sp___9 | Un amigo o miembro de su familia es desalojado o amenazado co desalojo<br>10 | leave_reason_sp___10 | Amenaza de abuso por pareja, miembro de la familia o otra persona

			11 leave_reason_sp___11 Ser dado de alta o el servici está siendo terminado<br>12 leave_reason_sp___12 Conflicto personal con los demás<br>13 leave_reason_sp___13 Otras preocupacione de salud o seguridad<br>14 leave_reason_sp___14 Otras violación(es) d renta<br>15 leave_reason_sp___15 Otro<br>99 leave_reason_sp___99 Prefiero no responder a esta pregunta
127	leave_reason_oth<br><br>Show the field ONLY if:<br>[leave_reason(14)] = '1' or [leave_reason(15)] = '1'	Please describe above answer.<br>*Please enter 99 if you choose not to answer this question.*	text, Required
128	leave_reason_oth_sp<br><br>Show the field ONLY if:<br>[leave_reason_sp(14)] = '1' or [leave_reason_sp(15)] = '1'	Por favor, describe la respuesta anterior.<br>*Por favor, escribe 99 si decide no responder a esta pregunta.*	text, Required
129	leave_when<br><br>Show the field ONLY if:<br>[stay_90d] = '0'	When do you need to leave? Please choose the one response that best matches your situation.	radio, Required<br>1 Today<br>2 2-3 days<br>3 Within the next 7 days<br>4 Within the next 2 weeks<br>5 Within the next 30 days<br>6 Within the next 60 days<br>7 Within the next 90 days<br>8 More than 90 days<br>9 Unsure<br>99 I choose not to answer this question

130	leave_when_sp<br><br>Show the field ONLY i f:<br>[stay_90d_sp] = '0'	¿Cuándo necesitas irte? Elija la respuesta que mejor se adapte a su situación.	radio, Required<br>1 Hoy<br>2 2-3 días<br>3 En los próximos 7 días<br>4 En las próximas 2 semanas<br>5 Dentro de los próximos 30 días<br>6 Dentro de los próximos 60 días<br>7 Dentro de los próximos 90 días<br>8 Más de 90 días<br>9 No estoy seguro<br>99 Prefiero no responder a esta pregunta
131	leave_where<br><br>Show the field ONLY i f:<br>[stay_90d] = '0'	Is there safe housing where you and your family can stay when you need to leave?	radio, Required<br>1 Yes<br>0 No<br>2 Unsure<br>99 I choose not to answer this question
132	leave_where_sp<br><br>Show the field ONLY i f:<br>[stay_90d_sp] = '0'	¿Existe una vivienda segura donde usted y su familia puedan quedarse cuando necesite irse?	radio, Required<br>1 Sí<br>0 No<br>2 No estoy seguro<br>99 Prefiero no responder a esta pregunta
133	leave_where_length<br><br>Show the field ONLY i f:<br>[leave_where] = '1'	How many nights can you stay in that place? Please choose the one response that best matches your situation.	radio, Required<br>1 Only one night<br>2 2-3 days<br>3 3-7 days<br>4 7-30 days<br>5 30-60 days<br>6 60-90 days<br>7 More than 90 days<br>8 Unsure<br>99 I choose not to answer this question

134	leave_where_length_sp<br>Show the field ONLY if:<br>[leave_where_sp] = '1'	¿Cuántas noches puedes quedarte en ese lugar? Elija la respuesta que mejor se adapte a su situación.	radio, Required<br>1 Solo una noche<br>2 2-3 días<br>3 3-7 días<br>4 7-30 días<br>5 30-60 días<br>6 60-90 días<br>7 Más de 90 días<br>8 No estoy seguro<br>99 Prefiero no responder a esta pregunta
135	move_help<br>Show the field ONLY if:<br>[surv_lang] = '1'	Do you need assistance finding some place to stay?	radio, Required<br>1 Yes<br>0 No<br>99 I choose not to answer this question
136	move_help_sp<br>Show the field ONLY if:<br>[surv_lang] = '2'	¿Necesitas ayuda para encontrar un lugar donde quedarte?	radio, Required<br>1 Sí<br>0 No<br>99 Prefiero no responder a esta pregunta
137	stay_help<br>Show the field ONLY if:<br>[stay_90d] = '0'	Do you need assistance to be able to stay at your current place?	radio, Required<br>1 Yes<br>0 No<br>99 I choose not to answer this question
138	stay_help_sp<br>Show the field ONLY if:<br>[stay_90d_sp] = '0'	¿Necesitas ayuda para poder quedarte en tu lugar actual?	radio, Required<br>1 Sí<br>0 No<br>99 Prefiero no responder a esta pregunta
139	sought_help<br>Show the field ONLY if:<br>[surv_lang] = '1'	Have you tried seeking housing resources such as a shelter, legal support, subsidized rent, a housing program or affordable housing in the last 12 months?<br>*If you have continuously lived in affordable housing or a housing program for the past 12 months and not sought any new resources, select "No."*	radio, Required<br>1 Yes<br>0 No<br>99 I choose not to answer this question

140	sought_help_sp<br><br>Show the field ONLY if:<br>[surv_lang] = '2'	¿Has intentado buscar recursos de vivienda, como un refugio, apoyo legal, renta subsidiada, un programa de vivienda o viviendas económicas en los últimos 12 meses?<br>*Si ha vivido continuamente en viviendas económicas o en un programa de vivienda durante los últimos 12 meses y no ha buscado ningún recurso nuevo, seleccione "No."*	radio, Required<br>1 Sí<br>0 No<br>99 Prefiero no responder a esta pregunta
141	help_type<br><br>Show the field ONLY if:<br>[sought_help] = '1'	If yes, what kind of housing resources did you seek out? Mark all that apply.	checkbox, Required<br>1 help_type___1 Legal support or advocacy services for tenants<br>2 help_type___2 Emergency shelter, including detox centers like Cherry Hill<br>3 help_type___3 Residential programs for survivors of domestic violence<br>4 help_type___4 Rent assistance<br>5 help_type___5 Transitional housing (stable housing that has a time limit of, for example, 6, 12, or 24 months)<br>6 help_type___6 Affordable housing (income-based, supportive, public, senior housing)<br>7 help_type___7 Other<br>99 help_type___99 I choose not to answer this question

142	help_type_sp<br>Show the field ONLY if:<br>[sought_help_sp] = '1'	Si la respuesta es sí, ¿qué tipo de recursos de vivienda buscó? Marque todo lo que corresponda.	checkbox, Required<br>1 | help_type_sp___1 | Apoyo legal o servicios de defensa para inquilinos/ocupantes<br>2 | help_type_sp___2 | Refugio de emergencia, que incluye centros de desintoxicación como Cherry Hill<br>3 | help_type_sp___3 | Programas residenciales para sobrevivientes de violencia doméstica<br>4 | help_type_sp___4 | Asistencia de renta<br>5 | help_type_sp___5 | Vivienda de transición (vivienda estable que tiene un límite de tiempo de, por ejemplo, 6, 12 o 24 meses)<br>6 | help_type_sp___6 | Vivienda económica (basada en los ingresos, de apoyo, pública, vivienda para personas mayores)<br>7 | help_type_sp___7 | Otro<br>99 | help_type_sp___99 | Prefiero no responder a esta pregunta
143	help_type_oth<br>Show the field ONLY if:<br>[help_type(7)] = '1'	Please describe "Other."<br>*Please enter 99 if you choose not to answer this question.*	notes, Required
144	help_type_oth_3<br>Show the field ONLY if:<br>[help_type_sp(7)] = '1'	Por favor describe "Otro."<br>*Por favor, escribe 99 si decide no responder a esta pregunta.*	notes, Required

145	found_help<br><br>Show the field ONLY if:<br>[sought_help] = '1'	Did you ultimately receive services from the resource(s) you sought out?	radio, Required<br>1 — Yes<br>2 — Yes, but it did not meet my needs<br>0 — No<br>99 — I choose not to answer this question
146	found_help_sp<br><br>Show the field ONLY if:<br>[sought_help_sp] = '1'	¿Recibió servicios del recurso(s) que buscó?	radio, Required<br>1 — Sí<br>2 — Sí, pero no satisfecho mis necesidades<br>0 — No<br>99 — Prefiero no responder a esta pregunta

147	not_helped<br><br>Show the field ONLY i f:<br>[found_help] = '0'	If no, why did you not receive services? Mark all that apply.	checkbox, Required<br>1 | not_helped___1 | I was told services were unavailable (i.e. no shelter beds, lost housing lottery)<br>2 | not_helped___2 | I was told I was ineligible<br>3 | not_helped___3 | I was put on a waitlist and never received services<br>4 | not_helped___4 | I could not complete application/ the application process was too burdensome<br>5 | not_helped___5 | I tried calling, but could not get through to anyone/ no one returned my call<br>6 | not_helped___6 | I completed all steps requested, but I never received linkage/ follow-up<br>7 | not_helped___7 | Language barriers prevented me from accessing the service<br>8 | not_helped___8 | I could not afford it/ meet the income requirements<br>9 | not_helped___9 | After attempting to access the resource, I chose not to use the service<br>10 | not_helped___10 | Other<br>99 | not_helped___99 | I choose not to answer this question
148	not_helped_sp	Si no, ¿por qué no recibió los servicios? Marque todo lo que corresponda.	checkbox, Required

	Show the field ONLY if:<br>[found_help_sp] = '0'		1	not_helped_sp___1	Me dijeron que los servicios no estaban disponibles (es decir, sin camas de refugio, lotería perdida de viviendas)
			2	not_helped_sp___2	Me dijeron que no era elegible
			3	not_helped_sp___3	Me pusieron en una lista de espera y nunca recibí servicios
			4	not_helped_sp___4	No pude completar la solicitud / el proceso de solicitud fue demasiado pesado
			5	not_helped_sp___5	Traté de llamar, pero no pude comunicarme con nadie/ nadie me devolvió la llamada
			6	not_helped_sp___6	Completé todos los pasos solicitados, pero nunca recibí enlace/ seguimiento
			7	not_helped_sp___7	Las barreras del idioma me impidieron acceder al servicio
			8	not_helped_sp___8	No podía pagarlo/ cumplir con los requisitos de ingresos

			9 | not_helped_sp___9 | Después de intentar acceder al recurso, elegí no usar el servicio<br>10 | not_helped_sp___10 | Otro<br>99 | not_helped_sp___99 | Prefiero no responder a esta pregunta
149	not_helped_oth<br><br>Show the field ONLY if:<br>[not_helped(10)] = '1'	Please describe "Other."<br>*Please enter 99 if you choose not to answer this question.*	notes, Required
150	not_helped_oth_sp<br><br>Show the field ONLY if:<br>[not_helped_sp(10)] = '1'	Por favor describe "Otro."<br>*Por favor, escribe 99 si decide no responder a esta pregunta.*	notes, Required
151	help_unused<br><br>Show the field ONLY if:<br>[not_helped(9)] = '1' or [found_help] = '2'	Why did the service not meet your needs, or why did you choose not to use it? Mark all that apply.	checkbox, Required<br>1 | help_unused___1 | I could not afford it/ was unable to meet the income requirements<br>2 | help_unused___2 | It required separation from family or pets<br>3 | help_unused___3 | I felt physically unsafe being in the space<br>4 | help_unused___4 | I felt that I was discriminated against based on identities I hold (i.e. race/ gender/ disability/ language/ age/ sexual orientation)<br>5 | help_unused___5 | The geographic location was too far from work/ school/ family/ my community

			6	help_unused___6	I felt unwelcome/ disrespected by staff
			7	help_unused___7	The program support ended, and I became homeless again
			8	help_unused___8	I was unable to securely store my personal belongings
			9	help_unused___9	I felt uncomfortable with religious elements of the program
			10	help_unused___10	I had to leave too early in the morning or be back too early at night
			11	help_unused___11	Other
			99	help_unused___99	I choose not to answer this question
152	help_unused_sp<br>Show the field ONLY i f:<br>[not_helped_sp(9)] = '1' or [found_help_sp] = '2'	¿Por qué el servicio no se ajusta a sus necesidades o por qué eligió no usarlo? Marque todo lo que corresponda.	checkbox, Required		
			1	help_unused_sp___1	No podía pagarlo/ no pude cumplir con los requisitos de ingresos
			2	help_unused_sp___2	Se requiere separación de la familia o mascotas
			3	help_unused_sp___3	Me sentía físicamente inseguro estar en el espacio

4	help_unused_sp___4	Sentí que fui discriminado en base a las identidades que tengo (es decir, raza/ género/ discapacidad/ idioma/ edad /orientación sexual)
5	help_unused_sp___5	La ubicación geográfica estaba demasiado lejos del trabajo/ escuela/ familia/ mi comunidad
6	help_unused_sp___6	Me sentí no bienvenido/ falta de respeto por el personal
7	help_unused_sp___7	El apoyo del programa terminó y me quedé sin hogar otra vez
8	help_unused_sp___8	No pude guardar de manera segura mis pertenencias personales
9	help_unused_sp___9	Me sentí incómodo con los elementos religiosos del programa
10	help_unused_sp___10	Tenía que irme temprano en la mañana o regresar demasiado temprano en la noche
11	help_unused_sp___11	Otro

			99 help_unused_sp___99 Prefiero no responder a esta pregunta
153	help_unused_oth<br><br>Show the field ONLY if: [help_unused(11)] = '1'	Please describe "Other."<br>*Please enter 99 if you choose not to answer this question.*	notes, Required
154	help_unused_oth_sp<br><br>Show the field ONLY if: [help_unused_sp(11)] = '1'	Por favor describe "Otro."<br>*Por favor, escribe 99 si decide no responder a esta pregunta.*	notes, Required
155	ed_house<br><br>Show the field ONLY if: [surv_lang] = '1'	Do you feel that you would benefit from having a housing specialist in the emergency department?	radio, Required<br>1 Yes<br>0 No<br>2 Unsure<br>99 I choose not to answer this question
156	ed_house_sp<br><br>Show the field ONLY if: [surv_lang] = '2'	¿Siente que se beneficiaría de tener un especialista en vivienda en el departamento de urgencias?	radio, Required<br>1 Sí<br>0 No<br>2 No estoy seguro<br>99 Prefiero no responder a esta pregunta
157	end<br><br>Show the field ONLY if: [surv_lang] = '1'	Please click the "Now" button on the right to record the current time.	text (datetime_seconds_mdy), Required
158	end_sp<br><br>Show the field ONLY if: [surv_lang] = '2'	Por favor haga clic en el botón "Now" a la derecha para registrar la hora actual.	text (datetime_seconds_mdy), Required
159	prapare_1st_complete	Section Header: *Form Status*<br>Complete?	dropdown<br>0 Incomplete<br>1 Unverified<br>2 Complete
Instrument: **AHC 1st** (ahc_1st)			
160	surv_lang_2	Which language would you like to take the survey in?	radio, Required<br>1 English<br>2 Spanish

161	ahc_start<br><br>Show the field ONLY if:<br>[surv_lang_2] = '1'	Section Header: *Housing Instability & Food Insecurity/ Inestabilidad de vivienda & Inseguridad alimentaria*<br><br>Please click the "Now" button on the right to record the current time.	text (datetime_seconds_mdy), Required<br>Custom alignment: RH
162	ahc_start_sp<br><br>Show the field ONLY if:<br>[surv_lang_2] = '2'	Por favor haga clic en el botón "Now" a la derecha para registrar la hora actual.	text (datetime_seconds_mdy), Required<br>Custom alignment: RH
163	house_tdy_ahc_2<br><br>Show the field ONLY if:<br>[surv_lang_2] = '1'	What is your housing situation today?	radio, Required<br>1 — I have a steady place to live.<br>2 — I have housing today, but I am worried about losing it in the future.<br>3 — I do not have a steady place to live (I am temporarily staying with others, in a hotel, in a shelter, living outside on the street, on a beach, in a car, abandoned building, bus or train station, or in a park).<br>99 — I choose not to answer this question.
164	house_tdy_ahc_sp_2<br><br>Show the field ONLY if:<br>[surv_lang_2] = '2'	¿Cuál es su situación de vivienda hoy?	radio, Required<br>1 — Tengo un lugar estable para vivir<br>2 — Tengo vivienda hoy, pero me preocupa perderla en el futuro<br>3 — No tengo un lugar estable para vivir (Me estoy quedando temporalmente con otros, en un hotel, en un refugio, viviendo afuera en la calle, en una playa, en un automóvil, en un edificio abandonado, en una estación de autobuses o de tren, o en un parque)<br>99 — Prefiero no responder a esta pregunta

165	house_problems_2<br><br>Show the field ONLY i f:<br>[surv_lang_2] = '1'	Think about the place you live. Do you have problems with any of the following? Select all that apply.	checkbox, Required<br>1 | house_problems_2___1 | Pests such as bugs, ants, or mice<br>2 | house_problems_2___2 | Mold<br>3 | house_problems_2___3 | Lead paint or pipes<br>4 | house_problems_2___4 | Lack of heat<br>5 | house_problems_2___5 | Oven or stove not working<br>6 | house_problems_2___6 | Smoke detectors missing or not working<br>7 | house_problems_2___7 | Water leaks<br>8 | house_problems_2___8 | None of the above<br>99 | house_problems_2___99 | I choose not to answer this question

166	house_problems_sp_2<br><br>Show the field ONLY i f:<br>[surv_lang_2] = '2'	Piensa en el lugar donde vives. ¿Tiene problemas con alguno de los siguientes? Marque todo lo que corresponda.	checkbox, Required<br>1 | house_problems_sp_2___1 | Plagas como insectos, hormigas o ratones<br>2 | house_problems_sp_2___2 | Molde<br>3 | house_problems_sp_2___3 | Pintura o tubos co plomo<br>4 | house_problems_sp_2___4 | Falta de calentón<br>5 | house_problems_sp_2___5 | Horno o estufa qu no funciona<br>6 | house_problems_sp_2___6 | Detectore de humo que falta o no funciona<br>7 | house_problems_sp_2___7 | Fugas de agua<br>8 | house_problems_sp_2___8 | Ninguna de las respuesta<br>99 | house_problems_sp_2___99 | Prefiero no responde a esta pregunta
167	ahc_food_text_2<br><br>Show the field ONLY i f:<br>[surv_lang_2] = '1'	Some people have made the following statements about their food situation. Please answer whether the statements were OFTEN, SOMETIMES, or NEVER true for you and your household in the last 12 months.	descriptive
168	ahc_food_text_sp_2<br><br>Show the field ONLY i f:<br>[surv_lang_2] = '2'	Algunas personas han hecho las siguientes declaraciones sobre su situación alimentaria. Por favor, responda si las declaraciones fueron FRECUENTES, A VECES o NUNCA verdad para usted y su hogar en los últimos 12 meses.	descriptive
169	food_worried_2<br><br>Show the field ONLY i f:<br>[surv_lang_2] = '1'	Within the past 12 months, you worried that your food would run out before you got money to buy more.	radio, Required<br>1 | Often true<br>2 | Sometimes true<br>3 | Never true<br>99 | I choose not to answer this question

170	food_worried_sp_2<br><br>Show the field ONLY if:<br>[surv_lang_2] = '2'	En los últimos 12 meses, le preocupaba que su comida se acabado antes de que tenga dinero para comprar más.	radio, Required<br>1 Con frecuencia<br>2 A veces<br>3 Nunca<br>99 Prefiero no responder a esta pregunta
171	food_ran_out_2<br><br>Show the field ONLY if:<br>[surv_lang_2] = '1'	Within the past 12 months, the food you bought just didn't last and you didn't have money to get more.	radio, Required<br>1 Often true<br>2 Sometimes true<br>3 Never true<br>99 I choose not to answer this question
172	food_ran_out_sp_2<br><br>Show the field ONLY if:<br>[surv_lang_2] = '2'	En los últimos 12 meses, la comida que compró simplemente no duró y no tenía dinero para obtener más.	radio, Required<br>1 Con frecuencia<br>2 A veces<br>3 Nunca<br>99 Prefiero no responder a esta pregunta
173	transport_ahc_2<br><br>Show the field ONLY if:<br>[surv_lang_2] = '1'	Section Header: *Transportation Needs, Utility Needs & Safety/ Necesidades de transporte, Necesidades de utilidad & La seguridad*<br><br>In the past 12 months, has lack of transportation kept you from medical appointments, meetings, work or from getting things needed for daily living?	radio, Required<br>1 Yes<br>0 No<br>99 I choose not to answer this question
174	transport_ahc_sp_2<br><br>Show the field ONLY if:<br>[surv_lang_2] = '2'	En los últimos 12 meses, ¿la falta de transporte lo mantuvo alejado de citas médicas, reuniones, trabajo o de obtener las cosas necesarias para la vida diaria?	radio, Required<br>1 Sí<br>0 No<br>99 Prefiero no responder a esta pregunta
175	utilities_threat_2<br><br>Show the field ONLY if:<br>[surv_lang_2] = '1'	In the past 12 months, has the electric, gas, oil, or water company threatened to shut off services in your home?	radio, Required<br>1 Yes<br>0 No<br>2 Already shut off<br>99 I choose not to answer this question
176	utilities_threat_sp_2<br><br>Show the field ONLY if:<br>[surv_lang_2] = '2'	En los últimos 12 meses, ¿la compañía de electricidad, gas, petróleo o agua ha amenazado con cerrar los servicios en su hogar?	radio, Required<br>1 Sí<br>0 No<br>2 Ya apagó<br>99 Prefiero no responder a esta pregunta

177	ahc_safety_text_2<br><br>Show the field ONLY if:<br>[surv_lang_2] = '1'	Because violence and abuse happen to a lot of people and affects their health, we are asking the following questions.	descriptive
178	ahc_safety_text_sp_2<br><br>Show the field ONLY if:<br>[surv_lang_2] = '2'	Debido a que la violencia y el abuso le ocurren a mucha gente y afectan su salud, nos hacemos las siguientes preguntas.	descriptive
179	abuse_phys_2<br><br>Show the field ONLY if:<br>[surv_lang_2] = '1'	How often does anyone, including family, physically hurt you?	radio, Required<br>1 Never<br>2 Rarely<br>3 Sometimes<br>4 Fairly often<br>5 Frequently<br>99 I choose not to answer this question
180	abuse_phys_sp_2<br><br>Show the field ONLY if:<br>[surv_lang_2] = '2'	¿Con qué frecuencia alguien, incluida la familia, lo lastima físicamente?	radio, Required<br>1 Nunca<br>2 Raramente<br>3 A veces<br>4 Bastante<br>5 Frecuentemente<br>99 Prefiero no responder a esta pregunta
181	abuse_belittle_2<br><br>Show the field ONLY if:<br>[surv_lang_2] = '1'	How often does anyone, including family, insult or talk down to you?	radio, Required<br>1 Never<br>2 Rarely<br>3 Sometimes<br>4 Fairly often<br>5 Frequently<br>99 I choose not to answer this question
182	abuse_belittle_sp_2<br><br>Show the field ONLY if:<br>[surv_lang_2] = '2'	¿Con qué frecuencia alguien, incluida la familia, le insulta o le critica?	radio, Required<br>1 Nunca<br>2 Raramente<br>3 A veces<br>4 Bastante<br>5 Frecuentemente<br>99 Prefiero no responder a esta pregunta

183	abuse_threats_2<br><br>Show the field ONLY i f:<br>[surv_lang_2] = '1'	How often does anyone, including family, threaten you with harm?	radio, Required<br>1 Never<br>2 Rarely<br>3 Sometimes<br>4 Fairly often<br>5 Frequently<br>99 I choose not to answer this question
184	abuse_threats_sp_2<br><br>Show the field ONLY i f:<br>[surv_lang_2] = '2'	¿Con qué frecuencia alguien, incluida la familia, lo amenaza con daño?	radio, Required<br>1 Nunca<br>2 Raramente<br>3 A veces<br>4 Bastante<br>5 Frecuentemente<br>99 Prefiero no responder a esta pregunta
185	abuse_curse_2<br><br>Show the field ONLY i f:<br>[surv_lang_2] = '1'	How often does anyone, including family, scream or curse at you?	radio, Required<br>1 Never<br>2 Rarely<br>3 Sometimes<br>4 Fairly often<br>5 Frequently<br>99 I choose not to answer this question
186	abuse_curse_sp_2<br><br>Show the field ONLY i f:<br>[surv_lang_2] = '2'	¿Con qué frecuencia alguien, incluida la familia, grita o maldice?	radio, Required<br>1 Nunca<br>2 Raramente<br>3 A veces<br>4 Bastante<br>5 Frecuentemente<br>99 Prefiero no responder a esta pregunta
187	decision_making_2<br><br>Show the field ONLY i f:<br>[surv_lang_2] = '1'	Section Header: *Disabilities & Substance Use/ Discapacidades & Uso de sustancias*<br><br>Because of a physical, mental, or emotional condition, do you have serious difficulty concentrating, remembering, or making decisions?	radio, Required<br>1 Yes<br>0 No<br>99 I choose not to answer this question

188	decision_making_sp_2<br><br>Show the field ONLY if:<br>[surv_lang_2] = '2'	Debido a una condición física, mental o emocional, ¿tiene serias dificultades para concentrarse, recordar o tomar decisiones?	radio, Required<br>1 Sí<br>0 No<br>99 Prefiero no responder a esta pregunta
189	daily_life_2<br><br>Show the field ONLY if:<br>[surv_lang_2] = '1'	Because of a physical, mental, or emotional condition, do you have difficulty doing errands alone such as visiting a doctor's office or shopping?	radio, Required<br>1 Yes<br>0 No<br>99 I choose not to answer this question
190	daily_life_sp_2<br><br>Show the field ONLY if:<br>[surv_lang_2] = '2'	Debido a una condición física, mental o emocional, ¿tiene dificultades para hacer mandados solo, como visitar el consultorio de un médico o ir de compras?	radio, Required<br>1 Sí<br>0 No<br>99 Prefiero no responder a esta pregunta
191	ahc_subs_use_text_2<br><br>Show the field ONLY if:<br>[surv_lang_2] = '1'	The next questions relate to your experience with alcohol, cigarettes, and other drugs. Some of the substances are prescribed by a doctor (like pain medications), but only count those if you have taken them for reasons or in doses other than prescribed. One question is about illicit or illegal drug use, but we only ask in order to identify community services that may be available to help you.	descriptive
192	ahc_subs_use_text_sp_2<br><br>Show the field ONLY if:<br>[surv_lang_2] = '2'	Las siguientes preguntas se relacionan con su experiencia con el alcohol, cigarrillos y otras drogas. Algunas de las sustancias son recetadas por un médico (como medicamentos para el dolor), pero solo cuéntelas si las ha tomado por razones o en dosis distintas a las recetadas. Una pregunta es acerca del uso ilícito o ilegal de drogas, pero solo preguntamos para identificar los servicios comunitarios que pueden estar disponibles para ayudarlo.	descriptive
193	etoh_2<br><br>Show the field ONLY if:<br>[surv_lang_2] = '1'	How many times in the past 12 months have you had 5 or more drinks in a day (males) or 4 or more drinks in a day (females)?<br>*One drink is 12 ounces of beer, 5 ounces of wine, or 1.5 ounces of 80-proof spirits.*	radio, Required<br>1 Never<br>2 Once or twice<br>3 Monthly<br>4 Weekly<br>5 Daily or almost daily<br>99 I choose not to answer this question

194	etoh_sp_2<br><br>Show the field ONLY if:<br>[surv_lang_2] = '2'	¿Cuántas veces en los últimos 12 meses ha bebido 5 o más bebidas en un día (hombres) o 4 o más bebidas en un día (mujeres)?<br>*Una bebida es 12 onzas de cerveza, 5 onzas de vino o 1.5 onzas de espíritus de 80 pruebas.*	radio, Required<br>1 Nunca<br>2 Una o dos veces<br>3 Mensual<br>4 Semanal<br>5 Diariamente o casi a diario<br>99 Prefiero no responder a esta pregunta
195	drugs_rx_2<br><br>Show the field ONLY if:<br>[surv_lang_2] = '1'	How many times in the past year have you used prescription drugs for non-medical reasons?	radio, Required<br>1 Never<br>2 Once or twice<br>3 Monthly<br>4 Weekly<br>5 Daily or almost daily<br>99 I choose not to answer this question
196	drugs_rx_sp_2<br><br>Show the field ONLY if:<br>[surv_lang_2] = '2'	¿Cuántas veces en el último año ha usado medicamentos recetados por razones no médicas?	radio, Required<br>1 Nunca<br>2 Una o dos veces<br>3 Mensual<br>4 Semanal<br>5 Diariamente o casi a diario<br>99 Prefiero no responder a esta pregunta
197	drugs_illegal_2<br><br>Show the field ONLY if:<br>[surv_lang_2] = '1'	How many times in the past year have you used illegal drugs?	radio, Required<br>1 Never<br>2 Once or twice<br>3 Monthly<br>4 Weekly<br>5 Daily or almost daily<br>99 I choose not to answer this question

198	drugs_illegal_sp_2<br><br>Show the field ONLY if:<br>[surv_lang_2] = '2'	¿Cuántas veces en el último año ha usado drogas ilegales?	radio, Required<br>1 Nunca<br>2 Una o dos veces<br>3 Mensual<br>4 Semanal<br>5 Diariamente o casi a diario<br>99 Prefiero no responder a esta pregunta
199	ahc_end_pra_start<br><br>Show the field ONLY if:<br>[surv_lang_2] = '1'	Please click the "Now" button on the right to record the current time.	text (datetime_seconds_mdy), Required<br>Custom alignment: RH
200	ahc_end_pra_start_sp<br><br>Show the field ONLY if:<br>[surv_lang_2] = '2'	Por favor haga clic en el botón "Now" a la derecha para registrar la hora actual.	text (datetime_seconds_mdy), Required<br>Custom alignment: RH
201	race_2<br><br>Show the field ONLY if:<br>[surv_lang_2] = '1'	Section Header: *Personal Characteristics/ Características Personales*<br><br>What is your race or ethnicity? Select all that apply.	checkbox, Required<br>11 race_2___11 Black/African American<br>12 race_2___12 White<br>13 race_2___13 Middle Eastern/North African<br>14 race_2___14 Hispanic, Latino or Spanish<br>15 race_2___15 Asian<br>16 race_2___16 Native Hawaiian<br>17 race_2___17 Pacific Islander<br>18 race_2___18 American Indian/Alaskan Native<br>19 race_2___19 Other<br>99 race_2___99 I choose not to answer this question

202	race_sp_2<br><br>Show the field ONLY i f:<br>[surv_lang_2] = '2'	¿Cuál es su raza o etnia? Marque todo lo que sea aplicable.	checkbox, Required<br>11 | race_sp_2___11 | Negro/ Afro Americano<br>12 | race_sp_2___12 | Blanco<br>13 | race_sp_2___13 | De Medio Oriente/ Norteafricano<br>14 | race_sp_2___14 | Hispano, latino o de origen español<br>15 | race_sp_2___15 | Asiático<br>16 | race_sp_2___16 | Nativo de Hawaii<br>17 | race_sp_2___17 | De las Islas del Pacifico<br>18 | race_sp_2___18 | Indio de los Estados Unidos/ Nativo de Alaska<br>19 | race_sp_2___19 | Otro<br>99 | race_sp_2___99 | Prefiero no responder a esta pregunta
203	race_oth_2<br><br>Show the field ONLY i f:<br>[race_2(19)] = '1' and [s urv_lang_2] = '1'	Please describe "Other."<br>*Please enter 99 if you choose not to answer this question.*	text, Required
204	race_oth_sp_2<br><br>Show the field ONLY i f:<br>[race_sp_2(19)] = '1'	Por favor describe "Otro."<br>*Por favor, escribe 99 si decide no responder a esta pregunta.*	text, Required
205	farm_work_2<br><br>Show the field ONLY i f:<br>[surv_lang_2] = '1'	At any point during the past 2 years, has seasonal or migrant farm work been your or your family's main source of income?	radio, Required<br>1 | Yes<br>0 | No<br>99 | I choose not to answer this question
206	farm_work_sp_2<br><br>Show the field ONLY i f:<br>[surv_lang_2] = '2'	En cualquier momento en los últimos 2 años, ¿el trabajo agrícola ha sido el ingreso principal de su familia?	radio, Required<br>1 | Sí<br>0 | No<br>99 | Prefiero no responder a esta pregunta

207	vet_2<br><br>Show the field ONLY i f:<br>[surv_lang_2] = '1'	Have you been discharged from the armed forces of the United States?	radio, Required<br>1 Yes<br>0 No<br>99 I choose not to answer this question
208	vet_sp_2<br><br>Show the field ONLY i f:<br>[surv_lang_2] = '2'	¿Ha servido en las fuerzas Armadas de los Estados Unidos?	radio, Required<br>1 Sí<br>0 No<br>99 Prefiero no responder a esta pregunta
209	lang_2<br><br>Show the field ONLY i f:<br>[surv_lang_2] = '1'	What language are you most comfortable speaking?	radio, Required<br>1 English<br>2 Spanish<br>3 Language other than English or Spanish<br>99 I choose not to answer this question
210	lang_sp_2<br><br>Show the field ONLY i f:<br>[surv_lang_2] = '2'	¿Con cuál idioma se siente más cómodo hablando?	radio<br>1 Inglés<br>2 Español<br>3 Idioma aparte del Inglés o Español<br>99 Prefiero no responder a esta pregunta
211	lang_oth_2<br><br>Show the field ONLY i f:<br>[lang_2] = '3' and [surv _lang_2] = '1'	Please describe "Other."<br>*Please enter 99 if you choose not to answer this question.*	text, Required<br>Custom alignment: RH
212	lang_oth_sp_2<br><br>Show the field ONLY i f:<br>[lang_sp_2] = '3'	Por favor describe "Otro."<br>*Por favor, escribe 99 si decide no responder a esta pregunta.*	text, Required
213	fam_home_2<br><br>Show the field ONLY i f:<br>[surv_lang_2] = '1'	Section Header: *Family & Home/ Familia y Hogar*<br>How many family members, including yourself, do you currently live with?<br>*Please enter 99 if you choose not to answer this question.*	text (integer), Required<br>Custom alignment: RH
214	fam_home_sp_2<br><br>Show the field ONLY i f:<br>[surv_lang_2] = '2'	¿Cuántos miembros de su familia principal viven con usted? (incluido usted mismo)<br>*Por favor, escribe 99 si decide no responder a esta pregunta.*	text (integer), Required<br>Custom alignment: RH

215	house_tdy_pra_2<br><br>Show the field ONLY i f:<br>[surv_lang_2] = '1'	What is your housing situation today?	radio, Required<br>1 I have housing<br>2 I do not have housing (staying with others, in a hotel, in a shelter, living outside on the street, on a beach, in a car, or in a park)<br>99 I choose not to answer this question
216	house_tdy_pra_sp_2<br><br>Show the field ONLY i f:<br>[surv_lang_2] = '2'	¿Cuál es su situación actualmente de su vivienda?	radio<br>1 Tengo vivienda<br>2 No tengo vivienda (viviendo con otros, en un hotel, en un albergue, viviendo en la calle, en una playa, en un carro, o en un parque)<br>99 Prefiero no responder a esta pregunta
217	house_worried_2<br><br>Show the field ONLY i f:<br>[surv_lang_2] = '1'	Are you worried about losing your housing?	radio, Required<br>1 Yes<br>0 No<br>2 N/A (not applicable)<br>99 I choose not to answer this question
218	house_worried_sp_2<br><br>Show the field ONLY i f:<br>[surv_lang_2] = '2'	¿Le preocupa que pudiera perder su vivienda?	radio, Required<br>1 Sí<br>0 No<br>2 N/A (no aplica)<br>99 Prefiero no responder a esta pregunta
219	street_2<br><br>Show the field ONLY i f:<br>[surv_lang_2] = '1'	What address do you live at?<br>*Street number and name. Please enter 99 if N/A or if you choose not to answer this question.*	text, Required, Identifier
220	street_sp_2<br><br>Show the field ONLY i f:<br>[surv_lang_2] = '2'	¿Cuál es su dirección de hogar?<br>*Número y nombre de la calle. Por favor, escribe 99 si no corresponde o si decide no responder a esta pregunta.*	text, Required, Identifier
221	city_state_2<br><br>Show the field ONLY i f:<br>[surv_lang_2] = '1'	City and state<br>*Please enter 99 if you choose not to answer this question.*	text, Required, Identifier

222	city_state_sp_2<br><br>Show the field ONLY i f:<br>[surv_lang_2] = '2'	Ciudad y estado<br>*Por favor, escribe 99 si decide no responder a esta pregunta.*	text, Required, Identifier
223	zip_code_2<br><br>Show the field ONLY i f:<br>[surv_lang_2] = '1'	Zip Code<br>*Please enter 99999 if N/A or if you choose not to answer this question.*	text (zipcode), Required, Identifier
224	zip_code_sp_2<br><br>Show the field ONLY i f:<br>[surv_lang_2] = '2'	Código postal<br>*Por favor, escribe 99 si no corresponde o si decide no responder a esta pregunta.*	text (zipcode), Required, Identifier
225	highest_edu_2<br><br>Show the field ONLY i f:<br>[surv_lang_2] = '1'	Section Header: *Money & Resources/ Dinero y Recursos*<br>What is the highest level of school that you have finished?	radio, Required<br>1 Less than a high school degree<br>2 High school diploma or GED<br>3 More than high school<br>99 I choose not to answer this question
226	highest_edu_sp_2<br><br>Show the field ONLY i f:<br>[surv_lang_2] = '2'	¿Cuál es el nivel escolar más alto que ha completado?	radio, Required<br>1 Escuela primaria<br>2 Preparatoria<br>3 Colegio, Universidad, o colegio técnico<br>99 Prefiero no responder a esta pregunta
227	work_now_2<br><br>Show the field ONLY i f:<br>[surv_lang_2] = '1'	What is your current work situation?	radio, Required<br>1 Unemployed and seeking work<br>2 Part-time or temporary work<br>3 Full-time work<br>4 Otherwise unemployed but not seeking work (ex. student, retired, disabled, unpaid primary caregiver)<br>99 I choose not to answer this question

228	work_now_sp_2<br><br>Show the field ONLY if:<br>[surv_lang_2] = '2'	¿Cuál es su situación laboral actualmente?	radio, Required<br>1 Desempleado y buscando empleo<br>2 Trabajo tiempo parcial o temporal (no agrícola)<br>3 Trabajo tiempo completo<br>4 Desempleado (ej. estudiante, jubilado, incapacitado, cuidador principal no remunerado)<br>99 Prefiero no responder a esta pregunta
229	work_oth_2<br><br>Show the field ONLY if:<br>[work_now_2] = '4'	Please describe above answer.<br>*Please enter 99 if you choose not to answer this question.*	text, Required
230	work_oth_sp_2<br><br>Show the field ONLY if:<br>[work_now_sp_2] = '4'	Por favor, describe la respuesta anterior.<br>*Por favor, escribe 99 si decide no responder a esta pregunta.*	text
231	insurance_2<br><br>Show the field ONLY if:<br>[surv_lang_2] = '1'	What is your main insurance?	radio, Required<br>1 None/ uninsured<br>2 Medicaid (Medi-Cal)<br>3 CHIP Medicaid<br>4 Medicare<br>5 Other public insurance (not CHIP)<br>6 Other public insurance (CHIP)<br>7 Private insurance<br>99 I choose not to answer this question
232	insurance_sp_2<br><br>Show the field ONLY if:<br>[surv_lang_2] = '2'	¿Cuál es su aseguranza médica?	radio, Required<br>1 Ninguno/ no asegurado<br>2 Medicaid (Medi-Cal)<br>3 CHIP Medicaid<br>4 Medicare<br>5 Otra aseguranza pública (No CHIP)<br>6 Otra aseguranza pública (CHIP)<br>7 Aseguranza privada<br>99 Prefiero no responder a esta pregunta

233	income_2<br><br>Show the field ONLY if:<br>[surv_lang_2] = '1'	During the past year, what was the total combined income for you and your family members you live with?<br>*This information will help us determine if you are eligible for any benefits. Please enter 99 if you choose not to answer this question.*	text (number), Required
234	income_sp_2<br><br>Show the field ONLY if:<br>[surv_lang_2] = '2'	¿Cuál fue el ingreso de su familia el año pasado?<br>*Esta información nos ayudará a determiner si usted califica para algún beneficio. Por favor, escribe 99 si decide no responder a esta pregunta.*	text (number), Required
235	needs_food_2	Section Header: *In the past year, have you or any family members you live with been unable to get any of the following when it was really needed? Select all that apply. Actualmente o el año pasado, ¿usted o alguna persona en su hogar tuvieron que privarse de algo que realmente se necesitaba? Marque todo lo que sea aplicable.*<br><br>Food/ Alimentos	radio (Matrix), Required<br>1 Yes/ Sí<br>2 No<br>3 I choose not to answer this question/ Prefiero no responder a esta pregunta
236	needs_utilities_2	Utilities/ Servicios públicos	radio (Matrix), Required<br>1 Yes/ Sí<br>2 No<br>3 I choose not to answer this question/ Prefiero no responder a esta pregunta
237	needs_med_2	Medicine or any healthcare (medical, dental, mental health, vision)/ Medicina o cualquier cuidado de salud (medico, dental, salud mental, vision)	radio (Matrix), Required<br>1 Yes/ Sí<br>2 No<br>3 I choose not to answer this question/ Prefiero no responder a esta pregunta
238	needs_phone_2	Phone/ Teléfono	radio (Matrix), Required<br>1 Yes/ Sí<br>2 No<br>3 I choose not to answer this question/ Prefiero no responder a esta pregunta
239	needs_clothing_2	Clothing/ Ropa	radio (Matrix), Required<br>1 Yes/ Sí<br>2 No<br>3 I choose not to answer this question/ Prefiero no responder a esta pregunta

240	needs_child_2	Child care/ Cuidado infantil	radio (Matrix), Required<br>1 Yes/ Sí<br>2 No<br>3 I choose not to answer this question/ Prefiero no responder a esta pregunta
241	needs_oth_2	Other/ Otro	radio (Matrix), Required<br>1 Yes/ Sí<br>2 No<br>3 I choose not to answer this question/ Prefiero no responder a esta pregunta
242	needs_oth_pra_2<br>Show the field ONLY if:<br>[needs_oth_2] = '1' and [surv_lang_2] = '1'	Please describe "Other."<br>*Please enter 99 if you choose not to answer this question.*	text, Required
243	needs_oth_pra_sp_2<br>Show the field ONLY if:<br>[needs_oth_2] = '1' and [surv_lang_2] = '2'	Por favor describe "Otro."<br>*Por favor, escribe 99 si decide no responder a esta pregunta.*	text, Required
244	transport_pra_2<br>Show the field ONLY if:<br>[surv_lang_2] = '1'	Has lack of transportation kept you from medical appointments, meetings, work, or from getting things needed for daily living? Select all that apply.	checkbox, Required<br>1 transport_pra_2___1 Yes, it has kept me from medical appointments or from getting my medications<br>2 transport_pra_2___2 Yes, it has kept me from non-medical meetings, appointments, work, or from getting things I need<br>0 transport_pra_2___0 No<br>99 transport_pra_2___99 I choose not to answer this question

245	transport_pra_sp_2<br>Show the field ONLY i f:<br>[surv_lang_2] = '2'	¿La falta de transportación le ha impedido ir a citas médicas, a reuniones, al trabajo, o conseguir cosas necesarias para la vida diaria? Marque todas las que aplican.	checkbox, Required<br>1 | transport_pra_sp_2___1 | Sí, me ha impedido ir citas médica o a recoger mis medicament<br>2 | transport_pra_sp_2___2 | Sí, me ha impedido ir reunions o citas no médicas, a trabajo, o conseguir cosas que necesito<br>0 | transport_pra_sp_2___0 | No<br>99 | transport_pra_sp_2___99 | Prefiero no responder a esta pregun
246	social_2<br>Show the field ONLY i f:<br>[surv_lang_2] = '1'	Section Header: *Social & Emotional Health/ Salud Social y Emocional*<br>How often do you see or talk to people that you care about and feel close to? (For example: talking to friends on the phone, visiting friends or family, going to church or club meetings)	radio, Required<br>1 | Less than once a week<br>2 | 1 or 2 times a week<br>3 | 3 to 5 times a week<br>4 | More than 5 times a week<br>99 | I choose not to answer this question
247	social_sp_2<br>Show the field ONLY i f:<br>[surv_lang_2] = '2'	¿Con qué frecuencia convive o conversa con personas por las que se preocupa y son cercanas a usted? (Por ejemplo: conversar con amigos por teléfono, visitar a amigos o familiares, asistir a la iglesia o reuniones)	radio, Required<br>1 | Menos de una vez por semana<br>2 | 1 o 2 veces por semana<br>3 | De 3 a 5 veces por semana<br>4 | Más de 5 veces por semana<br>99 | Prefiero no responder a esta pregunta
248	stress_2<br>Show the field ONLY i f:<br>[surv_lang_2] = '1'	Stress is when someone feels tense, nervous, anxious, or can't sleep at night because their mind is troubled. How stressed are you?	radio, Required<br>1 | Not at all<br>2 | A little bit<br>3 | Somewhat<br>4 | Quite a bit<br>5 | Very much<br>99 | I choose not to answer this question

249	stress_sp_2<br>Show the field ONLY if:<br>[surv_lang_2] = '2'	Estrés es cuando alguien se siente tenso, nervioso o no puede dormir en la noche porque su mente está preocupada. ¿Usted se siente estresado?	radio, Required<br>1 Para nada<br>2 Un poquito<br>3 Algunas veces<br>4 Bastante<br>5 Mucho<br>99 Prefiero no responder a esta pregunta
250	jail_2<br>Show the field ONLY if:<br>[surv_lang_2] = '1'	In the past year, have you spent more than 2 nights in a row in a jail, prison, detention center, or juvenile correction facility?	radio, Required<br>1 Yes<br>0 No<br>99 I choose not to answer this question
251	jail_sp_2<br>Show the field ONLY if:<br>[surv_lang_2] = '2'	En el último año, ¿ha pasado más de 2 noches seguidas en una cárcel, una prisión, un centro de detención, o en un centro correccional juvenil?	radio, Required<br>1 Sí<br>0 No<br>99 Prefiero no responder a esta pregunta
252	pra_end_house_start<br>Show the field ONLY if:<br>[surv_lang_2] = '1'	Please click the "Now" button on the right to record the current time.	text (datetime_seconds_mdy), Required
253	pra_end_house_start_sp<br>Show the field ONLY if:<br>[surv_lang_2] = '2'	Por favor haga clic en el botón "Now" a la derecha para registrar la hora actual.	text (datetime_seconds_mdy), Required
254	drugs_inj_2<br>Show the field ONLY if:<br>[surv_lang_2] = '1'	Have you ever used any drug by injection (non-medical use only)?	radio, Required<br>1 Yes<br>0 No<br>99 I choose not to answer this question
255	drugs_inj_sp_2<br>Show the field ONLY if:<br>[surv_lang_2] = '2'	¿Alguna vez ha usado algún medicamento por inyección (solo para uso no médico)?	radio, Required<br>1 Sí<br>0 No<br>99 Prefiero no responder a esta pregunta

256	drugs_inj_when_2<br><br>Show the field ONLY i f:<br>[drugs_inj_2] = '1'	When was the last time you injected?	radio, Required<br>1 In the past 90 days<br>2 In the past year<br>3 Over a year ago<br>99 I choose not to answer this question
257	drugs_inj_when_sp_2<br><br>Show the field ONLY i f:<br>[drugs_inj_sp_2] = '1'	¿Cuándo fue la última vez que le inyectaron a usted?	radio, Required<br>1 En los últimos 90 días<br>2 En el año pasado<br>3 Hace más de un año<br>99 Prefiero no responder a esta pregunta
258	eng_ability_2<br><br>Show the field ONLY i f:<br>[surv_lang_2] = '1'	Section Header: *Final Questions - Housing Focus/ Preguntas finales - Enfoque en la vivienda*<br><br>How well do you speak English?	radio, Required<br>1 Very well<br>2 Well<br>3 Not well<br>4 Not at all<br>99 I choose not to answer this question
259	eng_ability_sp_2<br><br>Show the field ONLY i f:<br>[surv_lang_2] = '2'	¿Que tan bien hablas ingles?	radio, Required<br>1 Muy bien<br>2 Bien<br>3 No muy bien<br>4 Mal<br>99 Prefiero no responder a esta pregunta
260	gender_2<br><br>Show the field ONLY i f:<br>[surv_lang_2] = '1'	What is your current gender identity?	radio, Required<br>1 Female/ Woman<br>2 Male/ Man<br>3 Trans male/ Trans man<br>4 Trans female/ Trans woman<br>5 Genderqueer/ Gender non-conforming<br>6 Different identity<br>99 I choose not to answer this question

261	gender_sp_2<br><br>Show the field ONLY if:<br>[surv_lang_2] = '2'	¿Cuál es su identidad preferencia de género actual?	radio, Required<br>1 Mujer<br>2 Hombre<br>3 Hombre trans<br>4 Mujer trans<br>5 Genderqueer/ Gender no conformes<br>6 Identidad diferente<br>99 Prefiero no responder a esta pregunta
262	moves_2<br><br>Show the field ONLY if:<br>[surv_lang_2] = '1'	How many times have you moved in the past 12 months?	radio, Required<br>0 0<br>1 1<br>2 2<br>3 3<br>4 4 or more<br>99 I choose not to answer this question
263	moves_sp_2<br><br>Show the field ONLY if:<br>[surv_lang_2] = '2'	¿Cuántas veces se ha mudado en los últimos 12 meses?	radio, Required<br>0 0<br>1 1<br>2 2<br>3 3<br>4 4 o más<br>99 Prefiero no responder a esta pregunta
264	house_unable_2<br><br>Show the field ONLY if:<br>[surv_lang_2] = '1'	During the last 12 months, was there a time when you or you and your family were not able to pay your mortgage, rent, or utility bills?	radio, Required<br>1 Yes<br>0 No<br>2 Unsure<br>3 I have been homeless<br>99 I choose not to answer this question
265	house_unable_sp_2<br><br>Show the field ONLY if:<br>[surv_lang_2] = '2'	Durante los últimos 12 meses, ¿hubo un momento en que usted o usted y su familia no pudieron pagar su hipoteca, renta o utilidades?	radio, Required<br>1 Sí<br>0 No<br>2 No estoy seguro<br>3 He estado sin hogar<br>99 Prefiero no responder a esta pregunta

266	needs_move_2<br><br>Show the field ONLY if:<br>[surv_lang_2] = '1'	During the last 12 months, did you or your children move in with other people even for a little while because you could not afford to pay your mortgage, rent, or utility bills?	radio, Required<br>1 Yes<br>0 No<br>2 Unsure<br>3 I have been homeless<br>99 I choose not to answer this question
267	needs_move_sp_2<br><br>Show the field ONLY if:<br>[surv_lang_2] = '2'	Durante los últimos 12 meses, ¿usted o sus hijos se mudaron con otras personas, incluso por un tiempo, porque no podía pagar su hipoteca, renta o utilidades?	radio, Required<br>1 Sí<br>0 No<br>2 No estoy seguro<br>3 He estado sin hogar<br>99 Prefiero no responder a esta pregunta

268	last_night_2<br><br>Show the field ONLY if:<br>[surv_lang_2] = '1'	Where did you stay last night? Please select the one response that best describes where you stayed last night.	radio, Required<br>1 Emergency shelter, including hotel or motel voucher paid for by a social service or charitable organization<br>2 Transitional housing for homeless persons<br>3 Permanent supportive housing for formerly homeless persons<br>4 Psychiatric hospital or other psychiatric facility<br>5 Substance abuse treatment facility or other detox facility<br>6 Hospital (non-psychiatric)<br>7 Jail, prison or juvenile detention facility<br>8 Half-way or three-quarter-way home for persons with criminal offenses<br>9 Room, apartment or house that you rent<br>10 Apartment or house that you own<br>11 In a friend's or family member's room, apartment or house<br>12 Hotel or motel paid for without emergency shelter voucher<br>13 Foster care home or foster care group home<br>14 Group home or other supervised residential care facility<br>15 Place not meant for human habitation (street, car, park, etc.)<br>16 Place or a situation that is dangerous to the health or safety of any household member<br>17 Other<br>99 I choose not to answer this question

269	last_night_sp_2<br><br>Show the field ONLY if:<br>[surv_lang_2] = '2'	¿Dónde te quedaste anoche? Seleccione la respuesta que mejor describa dónde se hospedó anoche.	radio, Required<br>1 Refugio de emergencia, incluyendo bono de hotel o motel pagado por un servicio social o organización benéfica<br>2 Vivienda de transición para personas sin hogar<br>3 Vivienda de apoyo permanente para personas que anteriormente no tenían hogar<br>4 Hospital psiquiátrico u otras facilidades psiquiátricas<br>5 Centro de tratamiento de abuso de sustancias o otras instalaciones de desintoxicación<br>6 Hospital (no psiquiátrico)<br>7 Cárcel, prisión o centro de detención juvenil<br>8 Hogar de transición para personas con ofensas criminales<br>9 Habitación, apartamento o casa que renta<br>10 Apartmento o casa que usted dueño de<br>11 En la habitación, apartamento o casa de un amigo o familiar<br>12 Hotel o motel pagado sin bono de refugio de emergencia<br>13 Hogar de cuidado de crianza o hogar de grupo de cuidado de crianza (foster care)<br>14 Hogar grupal o otro centro residencial de cuidado supervisado<br>15 Lugar no destinado a la habitación humana (calle, coche, parque, etc.)<br>16 Lugar o una situación que es peligrosa para la salud o la seguridad de cualquier miembro del hogar<br>17 Otro<br>99 Prefiero no responder a esta pregunta
270	last_night_oth_2<br><br>Show the field ONLY if:<br>[last_night_2] = '17'	Please describe "Other."<br>*Please enter 99 if you choose not to answer this question.*	text, Required

271	last_night_oth_sp_2<br><br>Show the field ONLY if:<br>[last_night_sp_2] = '17'	Por favor describe "Otro."<br>*Por favor, escribe 99 si decide no responder a esta pregunta.*	text, Required
272	last_night_length_2<br><br>Show the field ONLY if:<br>[surv_lang_2] = '1'	How long have you stayed in the place you stayed last night?	radio, Required<br>1 One week or less<br>2 More than one week, but less than one month<br>3 One to three months<br>4 More than three months, but less than one year<br>5 One year or longer<br>99 I choose not to answer this question
273	last_night_length_sp_2<br><br>Show the field ONLY if:<br>[surv_lang_2] = '2'	¿Cuánto tiempo hace que te quedaste en el lugar donde te hospedaste anoche?	radio, Required<br>1 Una semana o menos<br>2 Más de una semana, pero menos de un mes<br>3 De uno a tres meses<br>4 Más de tres meses, pero menos de un año<br>5 Un año o más<br>99 Prefiero no responder a esta pregunta
274	stay_90d_2<br><br>Show the field ONLY if:<br>[surv_lang_2] = '1'	Are you able to stay in this place for more than 90 days?	radio, Required<br>1 Yes<br>0 No/ Unsure<br>2 I'm currently homeless<br>99 I choose not to answer this question
275	stay_90d_sp_2<br><br>Show the field ONLY if:<br>[surv_lang_2] = '2'	¿Puedes quedarte en este lugar por más de 90 días?	radio, Required<br>1 Sí<br>0 No/ No estoy seguro<br>2 Estoy sin hogar<br>99 Prefiero no responder a esta pregunta
276	leave_reason_2<br><br>Show the field ONLY if:<br>[stay_90d_2] = '0'	Why do you need or want to leave? Please select all of the reasons why you need to leave the place you stayed last night.	checkbox, Required<br>1 leave_reason_2___1 Received an eviction notice

2	leave_reason_2___2	Non-payment of rent or past due rent
3	leave_reason_2___3	Unable to pay future rent because lost housing subsidy, job, or other income source
4	leave_reason_2___4	Non-payment of utilities or utility shut-off
5	leave_reason_2___5	Overcrowding
6	leave_reason_2___6	Inability to contribute to household costs
7	leave_reason_2___7	Housekeeping concerns (failure to maintain cleanliness of the unit)
8	leave_reason_2___8	Housing is or will be condemned
9	leave_reason_2___9	Friend or family member being evicted or threatened with eviction
10	leave_reason_2___10	Threat of abuse by partner, family member, or other
11	leave_reason_2___11	Being discharged or service is being terminated
12	leave_reason_2___12	Personal conflict with others
13	leave_reason_2___13	Other health or safety concerns

14	leave_reason_2___14	Other lease violation(s)
15	leave_reason_2___15	Other
99	leave_reason_2___99	I choose not to answer this question

277	leave_reason_sp_2<br>Show the field ONLY i f:<br>[stay_90d_sp_2] = '0'	¿Por qué necesitas o quieres irte? Por favor, marque todos los motivos por los que debe abandonar el lugar donde se alojó anoche.	checkbox, Required

1	leave_reason_sp_2___1	Recibió un aviso de desalojo
2	leave_reason_sp_2___2	Falta de pag de renta o renta vencid
3	leave_reason_sp_2___3	No se puede pagar la ren futura porq se perdió el subsidio de vivienda, el trabajo o otr fuente de ingresos
4	leave_reason_sp_2___4	Falta de pag de las utilidades o corte de utilidades
5	leave_reason_sp_2___5	Superpoblac
6	leave_reason_sp_2___6	No poder contribuir a l costos del hogar
7	leave_reason_sp_2___7	Problemas d limpieza (falt de mantenimie de la limpiez de la unidad)
8	leave_reason_sp_2___8	La vivienda e será declara en ruinas
9	leave_reason_sp_2___9	Un amigo o miembro de familia es desalojado o amenazado d desalojo

			10	leave_reason_sp_2___10	Amenaza de abuso por pareja, miembro de familia o otra persona
			11	leave_reason_sp_2___11	Ser dado de alta o el servi está siendo terminado
			12	leave_reason_sp_2___12	Conflicto personal con los demás
			13	leave_reason_sp_2___13	Otras preocupacion de salud o seguridad
			14	leave_reason_sp_2___14	Otras violación(es) renta
			15	leave_reason_sp_2___15	Otro
			99	leave_reason_sp_2___99	Prefiero no responder a esta pregunta
278	leave_reason_oth_2<br>Show the field ONLY if: [leave_reason_2(14)] = '1' or [leave_reason_2(15)] = '1'	Please describe above answer.<br>*Please enter 99 if you choose not to answer this question.*	text, Required		
279	leave_reason_oth_sp_2<br>Show the field ONLY if: [leave_reason_sp_2(14)] = '1' or [leave_reason_sp_2(15)] = '1'	Por favor, describe la respuesta anterior.<br>*Por favor, escribe 99 si decide no responder a esta pregunta.*	text, Required		

280	leave_when_2<br><br>Show the field ONLY if:<br>[stay_90d_2] = '0'	When do you need to leave? Please choose the one response that best matches your situation.	radio, Required<br>1 Today<br>2 2-3 days<br>3 Within the next 7 days<br>4 Within the next 2 weeks<br>5 Within the next 30 days<br>6 Within the next 60 days<br>7 Within the next 90 days<br>8 More than 90 days<br>9 Unsure<br>99 I choose not to answer this question
281	leave_when_sp_2<br><br>Show the field ONLY if:<br>[stay_90d_sp_2] = '0'	¿Cuándo necesitas irte? Elija la respuesta que mejor se adapte a su situación.	radio, Required<br>1 Hoy<br>2 2-3 días<br>3 En los próximos 7 días<br>4 En las próximas 2 semanas<br>5 Dentro de los próximos 30 días<br>6 Dentro de los próximos 60 días<br>7 Dentro de los próximos 90 días<br>8 Más de 90 días<br>9 No estoy seguro<br>99 Prefiero no responder a esta pregunta
282	leave_where_2<br><br>Show the field ONLY if:<br>[stay_90d_2] = '0'	Is there safe housing where you and your family can stay when you need to leave?	radio, Required<br>1 Yes<br>0 No<br>2 Unsure<br>99 I choose not to answer this question
283	leave_where_sp_2<br><br>Show the field ONLY if:<br>[stay_90d_sp_2] = '0'	¿Existe una vivienda segura donde usted y su familia puedan quedarse cuando necesite irse?	radio, Required<br>1 Sí<br>0 No<br>2 No estoy seguro<br>99 Prefiero no responder a esta pregunta

284	leave_where_length_2<br>Show the field ONLY if:<br>[leave_where_2] = '1'	How many nights can you stay in that place? Please choose the one response that best matches your situation.	radio, Required<br>1 Only one night<br>2 2-3 days<br>3 3-7 days<br>4 7-30 days<br>5 30-60 days<br>6 60-90 days<br>7 More than 90 days<br>8 Unsure<br>99 I choose not to answer this question
285	leave_where_length_sp_2<br>Show the field ONLY if:<br>[leave_where_sp_2] = '1'	¿Cuántas noches puedes quedarte en ese lugar? Elija la respuesta que mejor se adapte a su situación.	radio, Required<br>1 Solo una noche<br>2 2-3 días<br>3 3-7 días<br>4 7-30 días<br>5 30-60 días<br>6 60-90 días<br>7 Más de 90 días<br>8 No estoy seguro<br>99 Prefiero no responder a esta pregunta
286	move_help_2<br>Show the field ONLY if:<br>[surv_lang_2] = '1'	Do you need assistance finding some place to stay?	radio, Required<br>1 Yes<br>0 No<br>99 I choose not to answer this question
287	move_help_sp_2<br>Show the field ONLY if:<br>[surv_lang_2] = '2'	¿Necesitas ayuda para encontrar un lugar donde quedarte?	radio, Required<br>1 Sí<br>0 No<br>99 Prefiero no responder a esta pregunta
288	stay_help_2<br>Show the field ONLY if:<br>[stay_90d_2] = '0'	Do you need assistance to be able to stay at your current place?	radio, Required<br>1 Yes<br>0 No<br>99 I choose not to answer this question

289	stay_help_sp_2<br><br>Show the field ONLY if:<br>[stay_90d_sp_2] = '0'	¿Necesitas ayuda para poder quedarte en tu lugar actual?	radio, Required<br>1 Sí<br>0 No<br>99 Prefiero no responder a esta pregunta
290	sought_help_2<br><br>Show the field ONLY if:<br>[surv_lang_2] = '1'	Have you tried seeking housing resources such as a shelter, legal support, subsidized rent, a housing program or affordable housing in the last 12 months?<br>*If you have continuously lived in affordable housing or a housing program for the past 12 months and not sought any new resources, select "No."*	radio, Required<br>1 Yes<br>0 No<br>99 I choose not to answer this question
291	sought_help_sp_2<br><br>Show the field ONLY if:<br>[surv_lang_2] = '2'	¿Has intentado buscar recursos de vivienda, como un refugio, apoyo legal, renta subsidiada, un programa de vivienda o viviendas económicas en los últimos 12 meses?<br>*Si ha vivido continuamente en viviendas económicas o en un programa de vivienda durante los últimos 12 meses y no ha buscado ningún recurso nuevo, seleccione "No."*	radio, Required<br>1 Sí<br>0 No<br>99 Prefiero no responder a esta pregunta

<table>
<tr><td>292</td><td>help_type_2

Show the field ONLY i f:
[sought_help_2] = '1'</td><td>If yes, what kind of housing resources did you seek out? Mark all that apply.</td><td>checkbox, Required
<table>
<tr><td>1</td><td>help_type_2___1</td><td>Legal support or advocacy services for tenants</td></tr>
<tr><td>2</td><td>help_type_2___2</td><td>Emergency shelter, including detox centers like Cherry Hill</td></tr>
<tr><td>3</td><td>help_type_2___3</td><td>Residential programs for survivors of domestic violence</td></tr>
<tr><td>4</td><td>help_type_2___4</td><td>Rent assistance</td></tr>
<tr><td>5</td><td>help_type_2___5</td><td>Transitional housing (stable housing that has a time limit of, for example, 6, 12, or 24 months)</td></tr>
<tr><td>6</td><td>help_type_2___6</td><td>Affordable housing (income-based, supportive, public, senior housing)</td></tr>
<tr><td>7</td><td>help_type_2___7</td><td>Other</td></tr>
<tr><td>99</td><td>help_type_2___99</td><td>I choose not to answer this question</td></tr>
</table></td></tr>
</table>

293	help_type_sp_2<br><br>Show the field ONLY i f:<br>[sought_help_sp_2] = '1'	Si la respuesta es sí, ¿qué tipo de recursos de vivienda buscó? Marque todo lo que corresponda.	checkbox, Required<br>1 | help_type_sp_2___1 | Apoyo legal o servicios de defensa para inquilinos/ocupantes<br>2 | help_type_sp_2___2 | Refugio de emergencia, que incluye centros de desintoxicación como Cherry Hill<br>3 | help_type_sp_2___3 | Programas residenciales para sobrevivientes de violencia doméstica<br>4 | help_type_sp_2___4 | Asistencia de renta<br>5 | help_type_sp_2___5 | Vivienda de transición (vivienda estable que tiene un límite de tiempo de, por ejemplo, 6, 12 o 24 meses)<br>6 | help_type_sp_2___6 | Vivienda económica (basada en los ingresos, de apoyo, pública, vivienda para personas mayores)<br>7 | help_type_sp_2___7 | Otro<br>99 | help_type_sp_2___99 | Prefiero no responder a esta pregunta
294	help_type_oth_2<br><br>Show the field ONLY i f:<br>[help_type_2(7)] = '1'	Please describe "Other."<br>*Please enter 99 if you choose not to answer this question.*	notes, Required
295	help_type_oth_3_2<br><br>Show the field ONLY i f:<br>[help_type_sp_2(7)] = '1'	Por favor describe "Otro."<br>*Por favor, escribe 99 si decide no responder a esta pregunta.*	notes, Required

296	found_help_2<br><br>Show the field ONLY if:<br>[sought_help_2] = '1'	Did you ultimately receive services from the resource(s) you sought out?	radio, Required<br>1 — Yes<br>2 — Yes, but it did not meet my needs<br>0 — No<br>99 — I choose not to answer this question
297	found_help_sp_2<br><br>Show the field ONLY if:<br>[sought_help_sp_2] = '1'	¿Recibió servicios del recurso(s) que buscó?	radio, Required<br>1 — Sí<br>2 — Sí, pero no satisfecho mis necesidades<br>0 — No<br>99 — Prefiero no responder a esta pregunta
298	not_helped_2<br><br>Show the field ONLY if:<br>[found_help_2] = '0'	If no, why did you not receive services? Mark all that apply.	checkbox, Required<br>1 — not_helped_2___1 — I was told services were unavailable (i.e. no shelter beds, lost housing lottery)<br>2 — not_helped_2___2 — I was told I was ineligible<br>3 — not_helped_2___3 — I was put on a waitlist and never received services<br>4 — not_helped_2___4 — I could not complete application/ the application process was too burdensome<br>5 — not_helped_2___5 — I tried calling, but could not get through to anyone/ no one returned my call<br>6 — not_helped_2___6 — I completed all steps requested, but I never received linkage/ follow-up

			7 | not_helped_2___7 | Language barriers prevented me from accessing the service<br>8 | not_helped_2___8 | I could not afford it/ meet the income requirements<br>9 | not_helped_2___9 | After attempting to access the resource, I chose not to use the service<br>10 | not_helped_2___10 | Other<br>99 | not_helped_2___99 | I choose not to answer this question
299	not_helped_sp_2<br>Show the field ONLY i f:<br>[found_help_sp_2] = '0'	Si no, ¿por qué no recibió los servicios? Marque todo lo que corresponda.	checkbox, Required<br>1 | not_helped_sp_2___1 | Me dijeron que los servicios no estaban disponibles (es decir, sin camas de refugio, lotería perdida de viviendas)<br>2 | not_helped_sp_2___2 | Me dijeron que no era elegible<br>3 | not_helped_sp_2___3 | Me pusieron en una lista de espera y nunca recibí servicios<br>4 | not_helped_sp_2___4 | No pude completar la solicitud / el proceso de solicitud fue demasiado pesado

			5 | not_helped_sp_2___5 | Traté de llamar, pero no pude comunicarme con nadie/ nadie me devolvió la llamada<br>6 | not_helped_sp_2___6 | Completé todos los pasos solicitados, pero nunca recibí enlace/ seguimiento<br>7 | not_helped_sp_2___7 | Las barreras del idioma me impidieron acceder al servicio<br>8 | not_helped_sp_2___8 | No podía pagarlo/ cumplir con los requisitos de ingresos<br>9 | not_helped_sp_2___9 | Después de intentar acceder al recurso, elegí no usar el servicio<br>10 | not_helped_sp_2___10 | Otro<br>99 | not_helped_sp_2___99 | Prefiero no responder a esta pregunta
300	not_helped_oth_2<br>Show the field ONLY if: [not_helped_2(10)] = '1'	Please describe "Other."<br>*Please enter 99 if you choose not to answer this question.*	notes, Required
301	not_helped_oth_sp_2<br>Show the field ONLY if: [not_helped_sp_2(10)] = '1'	Por favor describe "Otro."<br>*Por favor, escribe 99 si decide no responder a esta pregunta.*	notes, Required
302	help_unused_2<br>Show the field ONLY if:	Why did the service not meet your needs, or why did you choose not to use it? Mark all that apply.	checkbox, Required

	[not_helped_2(9)] = '1' or [found_help_2] = '2'		

1	help_unused_2___1	I could not afford it/ was unable to meet the income requirements
2	help_unused_2___2	It required separation from family or pets
3	help_unused_2___3	I felt physically unsafe being in the space
4	help_unused_2___4	I felt that I was discriminated against based on identities I hold (i.e. race/ gender/ disability/ language/ age/ sexual orientation)
5	help_unused_2___5	The geographic location was too far from work/ school/ family/ my community
6	help_unused_2___6	I felt unwelcome/ disrespected by staff
7	help_unused_2___7	The program support ended, and I became homeless again
8	help_unused_2___8	I was unable to securely store my personal belongings
9	help_unused_2___9	I felt uncomfortable with religious elements of the program

#	Variable / Field Name	Field Label	Code	Choice Variable	Choice Label
			10	help_unused_2___10	I had to leave too early in the morning or be back too early at night
			11	help_unused_2___11	Other
			99	help_unused_2___99	I choose not to answer this question
303	help_unused_sp_2<br><br>Show the field ONLY if: [not_helped_sp_2(9)] = '1' or [found_help_sp_2] = '2'	¿Por qué el servicio no se ajusta a sus necesidades o por qué eligió no usarlo? Marque todo lo que corresponda.	checkbox, Required		
			1	help_unused_sp_2___1	No podía pagarlo/ no pude cumplir con los requisitos de ingresos
			2	help_unused_sp_2___2	Se requiere separación de la familia o mascotas
			3	help_unused_sp_2___3	Me sentía físicamente inseguro estar en el espacio
			4	help_unused_sp_2___4	Sentí que fui discriminado en base a las identidades que tengo (es decir, raza/ género/ discapacidad/ idioma/ edad /orientación sexual)
			5	help_unused_sp_2___5	La ubicación geográfica estaba demasiado lejos del trabajo/ escuela/ familia/ mi comunidad
			6	help_unused_sp_2___6	Me sentí no bienvenido/ falta de respeto por el personal

7	help_unused_sp_2___7	El apoyo del programa terminó y me quedé sin hogar otra vez
8	help_unused_sp_2___8	No pude guardar de manera segura mis pertenencias personales
9	help_unused_sp_2___9	Me sentí incómodo con los elementos religiosos del programa
10	help_unused_sp_2___10	Tenía que irme temprano en la mañana o regresar demasiado temprano en la noche
11	help_unused_sp_2___11	Otro
99	help_unused_sp_2___99	Prefiero no responder a esta pregunta

304	help_unused_oth_2<br>Show the field ONLY if:<br>[help_unused_2(11)] = '1'	Please describe "Other."<br>*Please enter 99 if you choose not to answer this question.*	notes, Required
305	help_unused_oth_sp_2<br>Show the field ONLY if:<br>[help_unused_sp_2(11)] = '1'	Por favor describe "Otro."<br>*Por favor, escribe 99 si decide no responder a esta pregunta.*	notes, Required
306	ed_house_2<br>Show the field ONLY if:<br>[surv_lang_2] = '1'	Do you feel that you would benefit from having a housing specialist in the emergency department?	radio, Required<br>1 Yes<br>0 No<br>2 Unsure<br>99 I choose not to answer this question

307	ed_house_sp_2<br><br>Show the field ONLY i f:<br>[surv_lang_2] = '2'	¿Siente que se beneficiaría de tener un especialista en vivienda en el departamento de urgencias?	radio, Required<br>1 Sí<br>0 No<br>2 No estoy seguro<br>99 Prefiero no responder a esta pregunta
308	end_2<br><br>Show the field ONLY i f:<br>[surv_lang_2] = '1'	Please click the "Now" button on the right to record the current time.	text (datetime_seconds_mdy), Required
309	end_sp_2<br><br>Show the field ONLY i f:<br>[surv_lang_2] = '2'	Por favor haga clic en el botón "Now" a la derecha para registrar la hora actual.	text (datetime_seconds_mdy), Required
310	ahc_1st_complete	Section Header: *Form Status*<br><br>Complete?	dropdown<br>0 Incomplete<br>1 Unverified<br>2 Complete
Instrument: **Conclusion** (conclusion)			
311	age	Age	text (integer, Min: 18, Max: 120), Required
312	sex	Gender according to Wellsoft	radio, Required<br>1 Female<br>2 Male
313	arrive_date	Patient's date of arrival	text (date_mdy), Required, Identifier<br>Custom alignment: RH
314	arrive_time	Patient's time of arrival<br>*First line in Wellsoft demographics, NOT Arrival (HIS). Military time; four numbers only with no colon.*	text (integer), Required<br>Custom alignment: RH
315	incomplete	If the survey was not administered, not completed in full in one sitting, or administered but not completed, please mark all reasons that apply.	checkbox, Required<br>1 incomplete___1 Patient declined<br>2 incomplete___2 Patient not coherent or lacking capacity to complete survey<br>3 incomplete___3 Patient stopped survey due to clinical team interruption<br>4 incomplete___4 Patient stopped survey and asked RA to return later

			5 | incomplete___5 | Patient stopped survey and did not agree to being re-approached<br>6 | incomplete___6 | Patient was interested, but declined to complete consent process<br>7 | incomplete___7 | Preferred language other than English or Spanish<br>12 | incomplete___12 | Clinician advised against approach<br>14 | incomplete___14 | Patient completed the survey a previous day<br>8 | incomplete___8 | Other<br>9 | incomplete___9 | Survey completed<br>13 | incomplete___13 | Survey completed, but did not initial<br>10 | incomplete___10 | Patient not approached (sleeping, discharged, LWOT, left AMA, etc) - for chart review only<br>11 | incomplete___11 | Unknown (pt not on screening log or screening outcome unclear) - for chart review only
316	incomplete_oth<br>Show the field ONLY if:<br>[incomplete(8)] = '1'	Please describe "Other."<br>*Please enter 99 if you choose not to answer this question.*	notes, Required
317	conclusion_complete	Section Header: *Form Status*<br>Complete?	dropdown<br>0 | Incomplete<br>1 | Unverified<br>2 | Complete

Instrument: **Chart Review** (chart_review)			
318	mrn	MRN	text (integer, Min: 0, Max: 999999999), Required, Identifier
319	esi	Acuity	text (integer, Min: 1, Max: 5), Required
320	custody	Was the patient in custody?	yesno, Required<br>1 Yes<br>0 No
321	psych	Was the patient on a psychiatric hold?	yesno, Required<br>1 Yes<br>0 No
322	lang_chart	Does the patient speak either English or Spanish?<br>*Look for preferred language in the Clinical Notes.*	radio, Required<br>1 Yes<br>0 No<br>99 Unknown
323	dispo	What was the patient's disposition?	radio, Required<br>1 Admit<br>2 Home/ Clinic at AHS<br>3 AMA/ Eloped<br>4 LWBS/ LWOT<br>5 Board & Care<br>6 Transfer to JGPH/ Herrick<br>7 Transfer - Other<br>8 Morgue<br>9 Law Enforcement<br>10 Other
324	dispo_oth<br><br>Show the field ONLY if:<br>[dispo] = '7' and [dispo] = '10'	Please describe "Other."<br>*Please enter 99 if you choose not to answer this question.*	text
325	dispo_date	Dispo date?	text (date_mdy)
326	dispo_time	Dispo time?<br><br>(What time was the dispo summary printed?)<br>*Military time; four numbers only with no colon.*	text
327	icd_1	Discharge ICD Code 1<br>*Go to the Chart tab, to the Dx/Instr section. Click on the field next to Diagnoses that lists the diagnoses. A pop-up with a table listing diagnoses and the corresponding ICD codes should appear. If not, press the spacebar, and this should cause the pop-up to appear. Just list the ICD code.*	text

328	icd_2	Discharge ICD Code 2<br>*Enter 99 if no second diagnosis provided.*	text
329	icd_3	Discharge ICD Code 3<br>*Enter 99 if no third diagnosis provided.*	text
330	ed_use	How many times has the patient been seen in EDs in the 12 months prior to and including this visit according to EDIE?<br>*If the patient has no EDIE tab, just enter the total number of visits to the Highland ED. Include the survey visit. Put 999 if pt LWBS or LWOT and no EDIE result.*	text (integer), Required
331	ed_highland	How many times has the patient been seen at Highland's ED in the 12 months prior to and including this visit?<br>*Enter the same number as above if the pt doesn't have an EDIE tab.*	text (integer), Required
332	admit_highland	How many ED visits at Highland Hospital resulted in an inpatient stay in the 12 months prior to and including this visit?	text (integer), Required
333	ed_other	How many times has the patient been seen at an ED outside of Highland Hospital in the 12 months prior to and including this visit?<br>*ENTER 999 (THREE 9s) IF PATIENT HAS NO SSN AND NO EDIE RESULTS. Also enter 999 if pt LWBS or LWOT.*	text (integer), Required
334	homeless	Are there any indicators that the patient is currently homeless? Check all that apply.	checkbox, Required<br>1 | homeless___1 | Yes, current homelessness noted in social history or chart notes<br>5 | homeless___5 | Yes, address field states pt is homeless/ has been left blank<br>2 | homeless___2 | Yes, address listed as 1411 East 31st Street<br>3 | homeless___3 | Other<br>4 | homeless___4 | None identified
335	homeless_oth<br><br>Show the field ONLY if:<br>[homeless(3)] = '1'	Please describe "Other."<br>*Please enter 99 if you choose not to answer this question.*	text

336	pmh	Does the patient have any of the following conditions in their past medical history?	checkbox, Required<br>1 pmh___1 Diabetes (DM)<br>2 pmh___2 Hypertension (HTN)<br>3 pmh___3 Heart disease (CHF, MI, CAD, any heart issues)<br>4 pmh___4 Stroke (CVA/TIA)<br>5 pmh___5 Chronic Obstructive Pulmonary Disease (COPD)<br>6 pmh___6 HIV<br>7 pmh___7 Cancer, in treatment<br>8 pmh___8 Cancer, history of<br>9 pmh___9 Mood disorders (anxiety, depression)<br>10 pmh___10 Schizophrenia<br>11 pmh___11 PTSD<br>12 pmh___12 Bipolar<br>13 pmh___13 Alcohol use disorder<br>14 pmh___14 Other drug use disorder (exclude marijuana)<br>15 pmh___15 None of the above<br>99 pmh___99 Missing/ No initials on authorization form
337	initial	Reviewer's initials	text, Required
338	initials_2	Second reviewer's initials, when applicable	text
339	chart_review_complete	Section Header: *Form Status*<br>Complete?	dropdown<br>0 Incomplete<br>1 Unverified<br>2 Complete
