# Supplementary figures and images for: Association of Social Needs and Housing Status Among Urban Emergency Department Patients
Source: West J Emerg Med. 2022 Oct 28;23(6):802–10. doi: 10.5811/westjem.2022.8.55705 (PMC9683759; doi:10.5811/westjem.2022.8.55705)

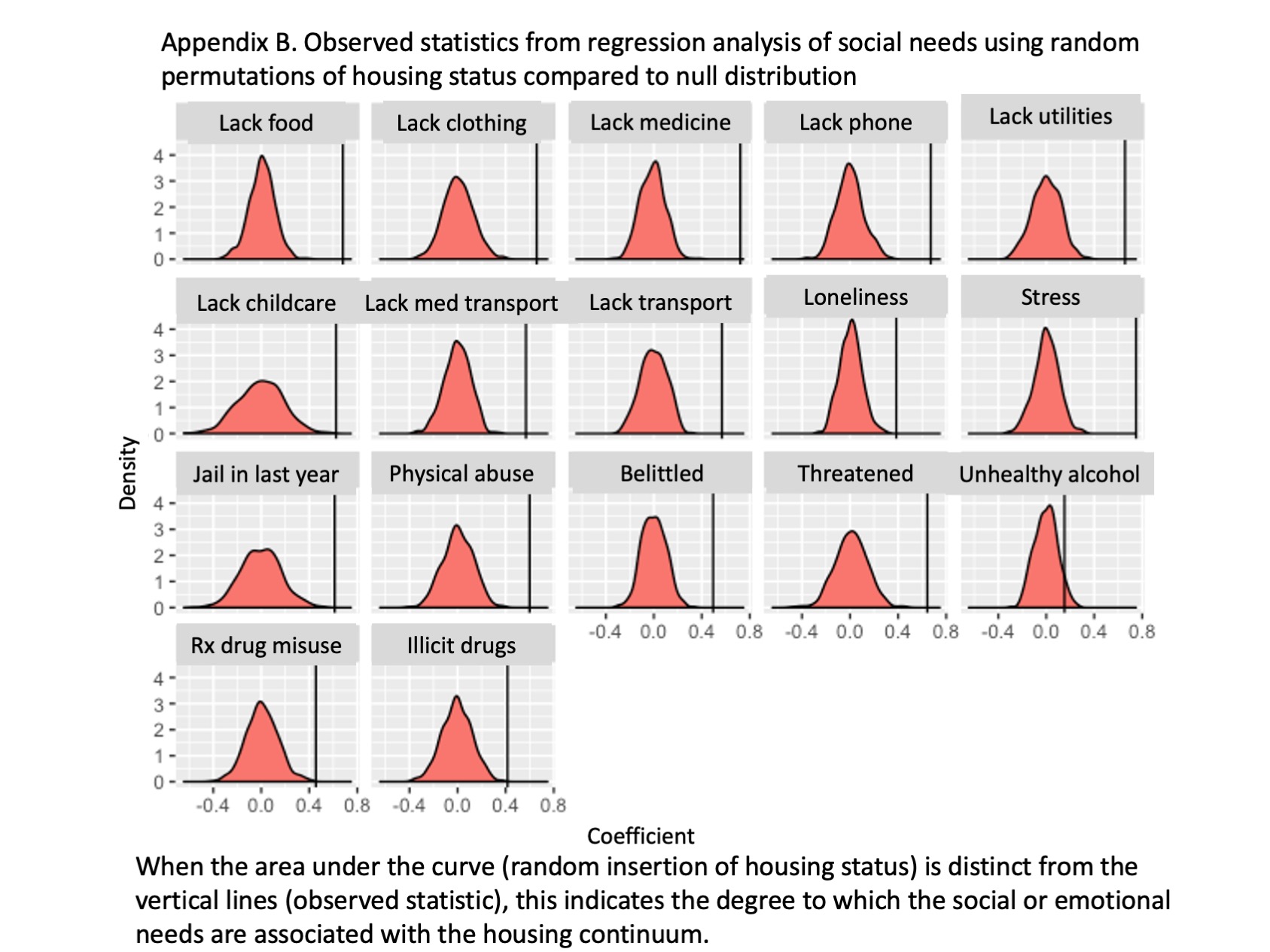

Supplement: Supplementary file 2 [file wjem-23-802-s002.jpg]
